# Field Emission Enhancement in Graphene/Cu Cathodes Through Laser‐Shock‐Induced Modulation of Geometry and Work Function

**DOI:** 10.1002/advs.78333

**Published:** 2026-11-10

**Authors:** Ximei Xu, Haonan Tao, Wenming Li, Xuyin Li, Jiacong Lin, Mingyang Yang, Ming Zhou

**Affiliations:** ^1^ State Key Laboratory of Clean and Efficient Turbomachinery Power Equipment Department of Mechanical Engineering Tsinghua University Beijing China; ^2^ Beijing Key Laboratory of Atomic‐Scale Precision Manufacturing and Cross‐Scale Device Fabrication Department of Mechanical Engineering Tsinghua University Beijing China; ^3^ Key Laboratory For Advanced Materials Processing Technology Ministry of Education Beijing China

**Keywords:** cold cathode, field emission, flexible vacuum electronics, graphene/metal interface, laser shock microforming, work function modulation

## Abstract

Cold cathodes enable compact, low‐power vacuum microelectronics with rapid activation. Graphene/metal cathodes offer a flexible, conductive platform, but efficient field emission requires local field concentration to coincide with reduced tunneling barriers while avoiding transfer contamination. Laser shock microforming converts graphene/Cu foils into flexible cathodes in one step without transfer while modulating the local work function. High‐curvature protrusions and gradients in graphene disorder, strain, Cu microstructure, surface chemistry, and graphene/Cu coupling create spatial variations in the local emission barrier. In individual monolayer graphene/Cu protrusions, these changes produce local work function contrasts reaching 0.12 eV and align lower barriers with highly curved regions. This spatial alignment enhances field emission, yielding a turn‐on field of 4.9 V µm^−1^, an emission current density of 2.2 mA cm^−2^, stable emission over 32 h, and durability after 3000 bending cycles. This work extends laser shock processing from geometric shaping to coupled structural and electronic barrier control in graphene/metal cold cathodes, providing a route that can be scaled over large areas for flexible vacuum electronics.

## Graphical abstract

Laser shock microforming converts graphene/Cu foils into flexible cathodes without transfer while modulating their local work function. Lower barriers near protrusion tops and rims coincide with electric field concentration, favoring electron emission. The cathodes exhibit enhanced field emission, stable operation for 32 h, and durability after 3000 bending cycles, offering a route to flexible vacuum electronics.

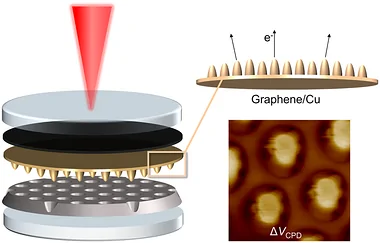

## 1 Introduction

Vacuum microelectronics is attracting renewed interest for applications requiring radiation resistance, operation at high frequencies and high power, as well as for emerging flexible vacuum devices such as compact X‐ray sources [1, 2, 3]. Cold cathode electron sources are key building blocks in such systems because electron emission without filament heating enables rapid activation with low power consumption and compact device integration [4]. Although traditional Spindt emitters based on silicon or metals have long served as benchmark electron sources [5], their use in flexible electronics and wearable X‐ray systems is strongly limited by the rigidity of silicon and the relatively high work function of conventional metals. Field emission is commonly described by Fowler–Nordheim (FN) tunneling [6, 7, 8], in which the emission current depends nonlinearly on the field enhancement factor (*β*) and the local work function (*Φ*_local_). Spatial variations in *Φ*_local_ and surface potential can further reshape the effective barrier [9, 10] and concentrate emission at only a small fraction of sites. Consequently, nanostructured carbon materials, especially graphene and carbon nanotubes, have emerged as leading candidates for next‐generation cathodes because they combine favorable emitter geometries with excellent mechanical robustness [11, 12, 13, 14, 15].

To date, performance optimization of nanostructured carbon cathodes has largely proceeded through trade‐offs between geometry and stability. Structures with high aspect ratios such as vertically aligned carbon nanotubes provide large *β* values and low turn‐on fields [11, 12], but cathodes can suffer from limited heat dissipation at the interface between the emitter and the substrate, which can lead to Joule heat accumulation and vacuum breakdown under high current [16]. Graphene provides an attractive platform because it combines high in‐plane conductivity, good mechanical strength, and an electronic structure that is sensitive to graphene/metal coupling [17, 18]. Graphene can be grown directly over large areas on Cu foils by chemical vapor deposition (CVD) [19, 20], yielding a transfer‐free graphene/Cu heterostructure that is both flexible and electrically conductive. Compared with flexible platforms based on polymer substrates, graphene/Cu foils offer robust current transport and heat dissipation and avoid constraints associated with outgassing that complicate operation at high voltage in vacuum. However, planar graphene films on metals usually lack sufficient geometric enhancement, whereas transferred graphene on patterned substrates often suffers from imperfect conformal contact as well as polymer residue and other interfacial contaminants introduced during transfer [21, 22, 23]. These factors can generate uncontrolled doping and spatially varying emission barriers, thereby obscuring the intrinsic emission behavior. Because *β* is primarily set by emitter geometry, whereas *Φ*_local_ is sensitive to graphene work function and graphene/metal interfacial charge transfer, the two parameters are usually optimized through separate structural and electronic routes. A central challenge, therefore, is to integrate three‐dimensional patterning of graphene on metal with control over the local emission barrier in a scalable process that does not require transfer.

Such a coupled strategy would ideally align regions of highest curvature with regions of lowest work function, thereby creating emission hot spots in which *β* and *Φ*_local_ act cooperatively to enhance electron tunneling. Because spatial variations in *Φ*_local_ and surface potential can reshape the effective barrier on the nanoscale, control over the spatial distribution of *Φ*_local_ is important for both emission uniformity and device durability. Laser shock microforming provides a distinctive route for this purpose. Unlike mechanical stamping under quasi‐static loading, laser shock generates transient pressures on the order of several gigapascals and drives confined plastic deformation at high strain rates [24]. Although laser shock has been used for surface hardening and metal forming [25, 26], and has been reported to alter the electronic structure of monolayer graphene through strain [27], whether shock loading can spatially modulate the local work function of graphene/metal heterostructures remains largely unexplored. Beyond geometric shaping, shock loading can introduce heterogeneous residual stress and microstructural refinement in Cu [28, 29], together with defect structures associated with deformation [30], which may perturb graphene/metal coupling and modulate *Φ*_local_ through changes in interfacial charge transfer and surface dipoles. In particular, it remains unclear how extreme strain rates of approximately 10^6^ s^−1^ affect the graphene/metal interface, whether variations in *Φ*_local_ induced by shock are colocalized with regions of high curvature, and whether the resulting strain and defect landscape can be harnessed to modulate the surface potential for field emission.

Here we demonstrate that laser shock microforming converts graphene/Cu foils into flexible field emission cathodes without transfer, using a single process to define protrusion geometry and modulate the local work function. During shock loading, deformation at high strain rates forms protrusion arrays and introduces spatial heterogeneity in graphene disorder and strain, Cu microstructure, surface chemical state, and graphene/Cu interfacial coupling. Cross‐sectional transmission electron microscopy (TEM), Raman spectroscopy, X‐ray photoelectron spectroscopy (XPS), and Kelvin probe force microscopy (KPFM), supported by density functional theory (DFT) calculations, show that these structural, chemical, and interfacial changes modulate the local emission barrier, producing local work function contrasts reaching 0.12 eV within individual monolayer graphene/Cu protrusions. Regions with lower barriers are located near the protrusion top and rim, where electrostatic simulations indicate strong local field concentration, providing a physical basis for emission hot spot formation. The resulting cathodes exhibit lower turn‐on fields, higher emission current densities, long‐term emission stability, and durability under bending. Overall, these results show that heterogeneity generated by shock can create self‐aligned emission sites in which emitter geometry and barrier modulation act together, positioning laser shock microforming as a scalable route to flexible graphene/metal cold cathodes for vacuum electronics.

## 2 Results and Discussion

### 2.1 Laser Shock Microforming of Graphene/Cu Cathodes

We used laser shock microforming with a rigid template to form controlled geometry and introduce spatially heterogeneous deformation in graphene/Cu foils (Figure 1a). The process stack combined a rigid template, a graphene/Cu foil, an absorption layer, and a transparent confinement layer. Patterning was carried out with a nano‐patterned sapphire substrate (NPSS) template and anodic aluminum oxide (AAO) templates. A pulsed neodymium‐doped yttrium aluminum garnet (Nd:YAG) nanosecond laser irradiated the graphite sacrificial coating to generate a confined plasma plume. Under confinement, plasma expansion produced a transient pressure pulse that drove deformation at high strain rates. Using the confined plasma model for laser‐induced plasmas in confined geometry [24] and actual processing parameters, we estimated a nominal pressure scale on the order of several gigapascals and a characteristic strain rate on the order of 10^6^–10^7^ s^−1^ (Note S1). This pressure pulse was sufficient to plastically deform the Cu foil, forcing the graphene/Cu foil to conform to the template and thereby imprinting the template topography onto the foil surface [25, 26]. Patterning over a large area was achieved by raster scanning with controlled spot overlap. After processing, the patterned region exhibited iridescent structural color arising from the periodic protrusion array, visible over a large area (Figure 1b). Atomic force microscopy (AFM) height profiles confirmed topographic replication from the template to the graphene/Cu surface (Figure 1c), while scanning electron microscopy (SEM) images showed periodic protrusion arrays formed using NPSS and AAO templates (Figure 1d–g).

**FIGURE 1:**
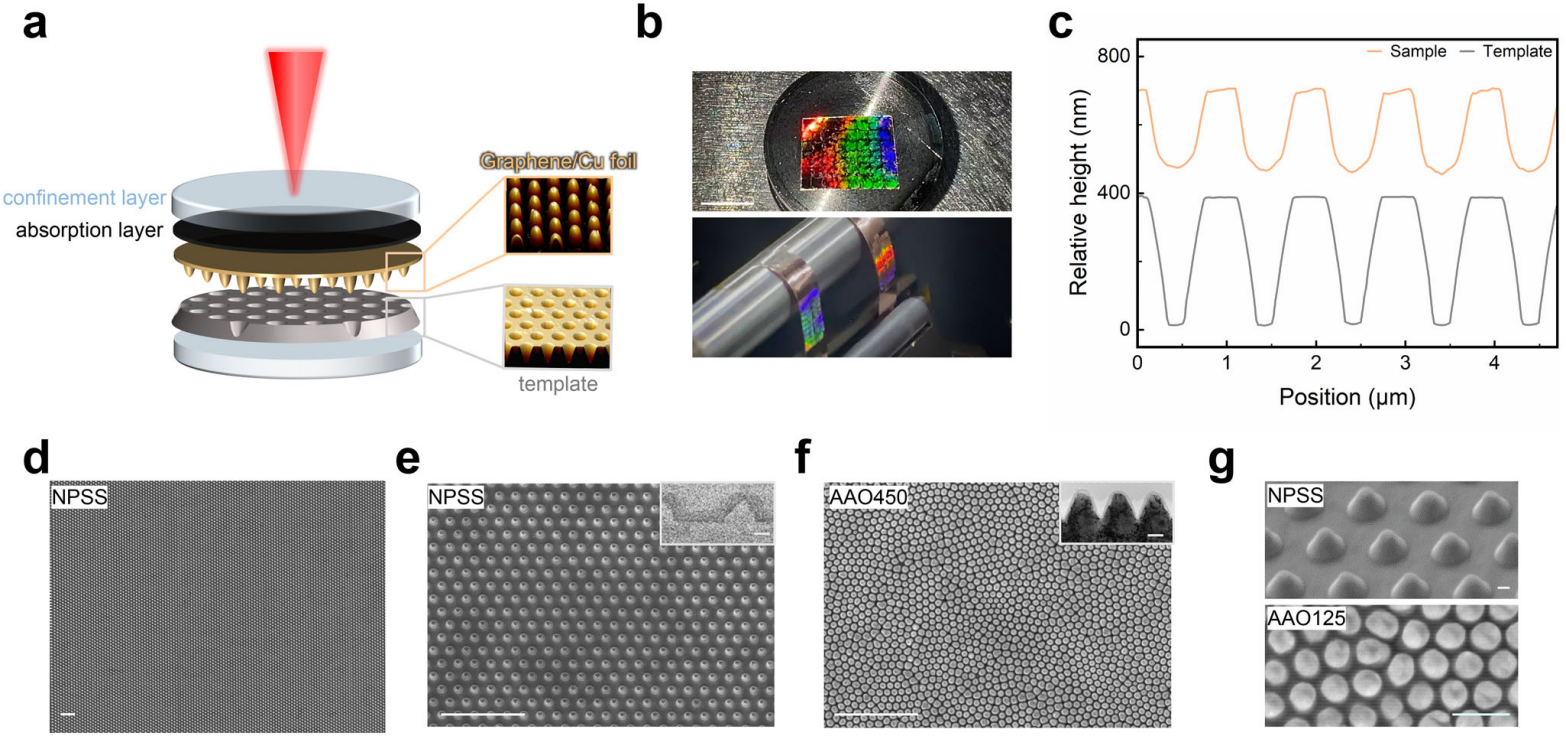
Laser shock microforming of graphene/Cu foils. (a) Schematic illustration of the laser shock microforming process used to fabricate periodic protrusion arrays in graphene/Cu foils. The process stack comprises a rigid template, a graphene/Cu foil, an absorption layer, and a transparent confinement layer. (b) Photographs of the processed graphene/Cu foil after laser shock, showing iridescent structural color arising from the periodic protrusion array in flat and curved configurations. Scale bar, 1 cm in the upper photograph. (c) Relative height profiles of the template and the replicated graphene/Cu surface, showing topographic imprinting after laser shock. The replicated surface profile is inverted to show its correspondence with the template profile. (d) Overview SEM image of a periodic protrusion array formed on graphene/Cu using an NPSS template. (e) SEM image of protrusion arrays formed using an NPSS template with a 1 µm period. The inset shows the corresponding cross‐sectional morphology of the graphene/Cu surface. (f) SEM image of protrusion arrays formed using an AAO template with a nominal feature size of 450 nm. The inset shows a representative cross‐sectional image of the replicated protrusions. (g) Higher magnification SEM images of protrusion arrays formed using NPSS and AAO templates with different feature sizes. Scale bars, 5 µm in (d–f) main images and 200 nm in the insets and (g). NPSS denotes nano‐patterned sapphire substrate, and AAO denotes anodic aluminum oxide.

The absorption layer separated the laser interaction zone from the sample and helped prevent direct laser ablation of the graphene/Cu foil. Within the processing window used here, no macroscopic peeling or delamination of graphene from the Cu substrate was observed across the patterned region. During the deformation, graphene remained attached to the Cu surface, as further supported by the Raman spectroscopy and XPS results presented below. Demolding was achieved without visible residue, consistent with graphene providing a relatively low shear interface between the template and the Cu surface. This transfer‐free process transforms graphene/Cu foils into three‐dimensional cathode surfaces while avoiding polymer residues and uncontrolled interfaces associated with transfer.

In the following sections, we examine how laser shock modifies the graphene/metal heterostructure, with emphasis on surface electronic states and their roles in modulating the local work function and field emission. Samples A (1‐p) and C (2‐p) represent pristine CVD monolayer and bilayer graphene on Cu, respectively, whereas samples B (1‐L) and D (2‐L) are the corresponding foils after laser shock. Unless otherwise specified, 1‐L and 2‐L refer to laser‐shocked samples processed with the NPSS template.

### 2.2 Shock‐Induced Structural and Chemical Heterogeneity

Cross‐sectional TEM of protrusions formed on monolayer graphene/Cu foils reveals strong spatial heterogeneity in the structure generated by laser shock. For clarity, we define four characteristic regions in the laser‐shocked graphene/Cu surface (Figure 2a): the protrusion top region, protrusion sidewall region, protrusion root region, and flat graphene/Cu substrate region. The graphene contrast changes abruptly at the boundary between the flat substrate region and the deformed protrusion (Figure 2b). In the flat substrate region, the carbon signal appears relatively continuous and uniform, which is consistent with monolayer graphene remaining largely intact and conformally covering the Cu surface, similar to the CVD graphene/Cu heterostructure before shock. An energy‐dispersive X‐ray spectroscopy (EDS) line scan shows an enhanced carbon signal localized near the top interface region (Figure S1). By contrast, graphene along the sidewall remains detectable but rarely appears as a continuous layer ordered over long ranges. Low‐voltage imaging at 60 kV confirms the same spatial trend: graphene is clearly visible in the flat substrate region, whereas graphene in the sidewall and root regions is strongly attenuated and appears locally fragmented (Figure S2). During forming, the Cu substrate plastically flows into the template cavity and drags the surface graphene. Along the sidewall region, direct contact between the graphene/Cu surface and the template is expected to impose stronger local compression and friction than in other regions. Interfacial sliding at the graphene/Cu interface and defect generation in graphene are therefore key contributors to the observed disorder.

**FIGURE 2:**
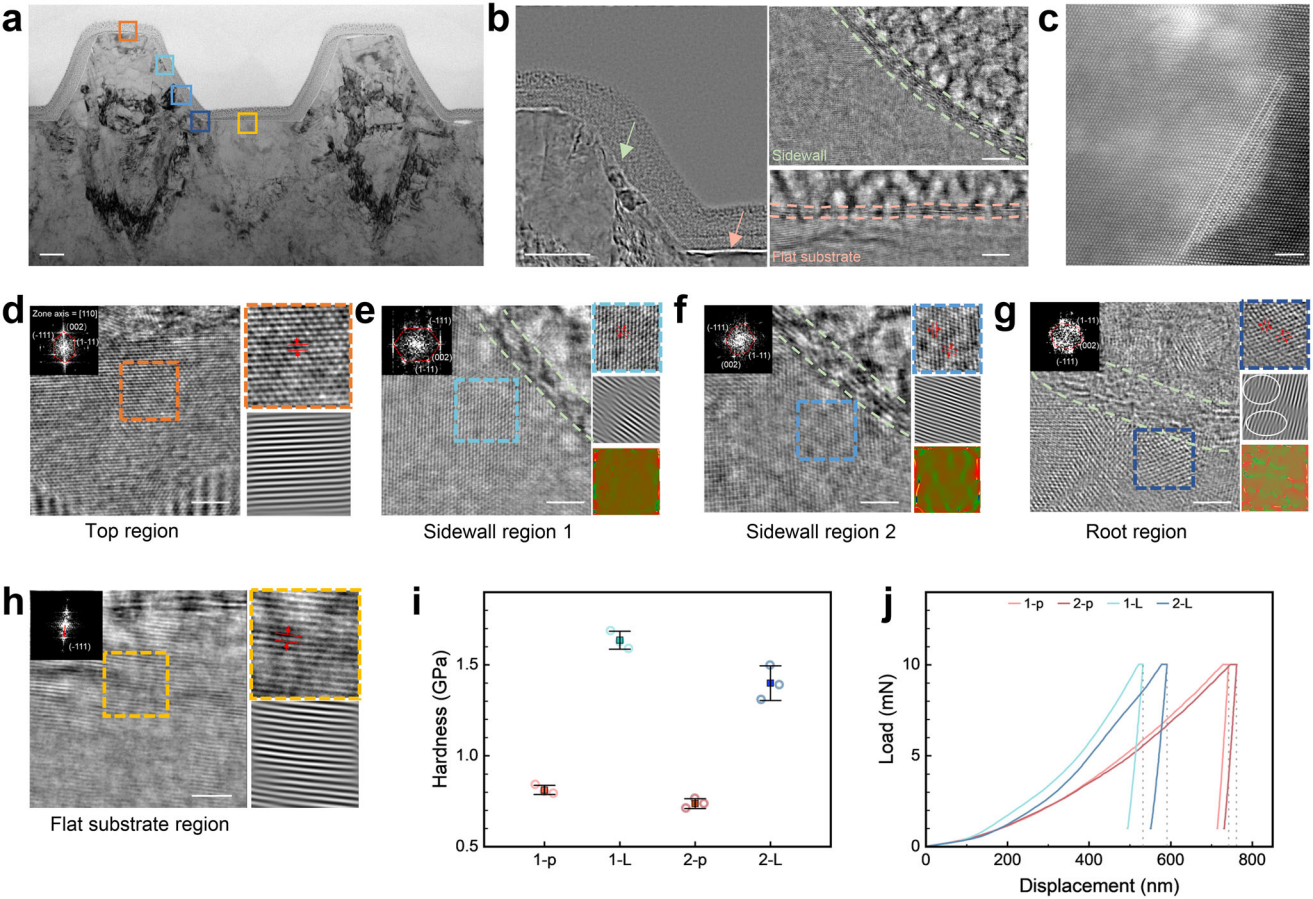
Cross‐sectional TEM analysis and nanoindentation of graphene/Cu protrusions after laser shock. (a) Cross‐sectional TEM image of protrusions formed on monolayer graphene/Cu, with colored boxes marking representative protrusion top, sidewall, root, and flat graphene/Cu substrate regions analyzed in (d–h). (b) Cross‐sectional overview image and high‐resolution TEM images of the sidewall and flat substrate regions, highlighting spatial changes in graphene continuity after deformation. (c) High‐resolution TEM image of the protrusion root region, showing stacking faults and related planar defects in Cu. (d–h) High‐resolution TEM images from the top region (d), sidewall region 1 (e), sidewall region 2 (f), root region (g), and flat substrate region (h) marked in (a). Insets show fast Fourier transform (FFT) patterns and Fourier‐filtered inverse FFT images; geometric phase analysis (GPA) maps are included in (e–g). These analyses reveal spatially heterogeneous grain refinement, planar defects, and lattice distortion after laser shock. (i) Statistical comparison of hardness, *H*, for pristine monolayer and bilayer graphene/Cu foils and their counterparts after laser shock. Open circles denote individual measurements, and squares and error bars denote means ± standard deviation (n = 3 independent indents). (j) Representative load–displacement curves for 1‐p, 1‐L, 2‐p and 2‐L measured under the same peak load, showing lower maximum indentation depths after laser shock. Scale bars, 100 nm in (a) and the overview image in (b), 5 nm in the high‐resolution images in (b), and 2 nm in (c–h).

The Cu microstructure after laser shock differs markedly from that of the Cu foil before processing, which contains coarse grains. High‐resolution images, together with FFT patterns calculated from selected regions, reveal substantial grain refinement and spatially heterogeneous nanocrystalline domains (Figure 2c–h). In the root region, where shear between the foil and the template is expected to be strongest, the refinement is most pronounced and is accompanied by stacking fault structures (Figure 2c,g). Numerous planar defect bundles aligned with specific crystallographic planes are also observed in Cu near the sidewall regions (Figure S3). These features are consistent with defects related to stacking faults generated during deformation at high strain rates. GPA reveals pronounced local lattice distortion in the sidewall and root regions (Figure 2e–g). By contrast, the grains in the top region are also refined, but to a lesser degree, possibly because local deformation there is dominated more by stretching associated with extrusion and material flow than by intense shear. The ultrafast loading intrinsic to laser shock drives microstructural evolution within a very short time window through intense dislocation activity and rapid rearrangement. For Cu, which has moderate stacking fault energy, plastic deformation is generally dominated by dislocation glide. The involvement of partial dislocations can leave behind stacking faults, and deformation twinning may also occur under the highest local shear stress and strain rate conditions [30]. Overall, laser shock produces coupled spatial gradients in graphene integrity, Cu defect density, and local lattice strain. These gradients provide a microstructural basis for surface potential variation and local work function modulation. In particular, graphene defects and interfacial sliding along the sidewall region can locally expose Cu and promote chemically and structurally heterogeneous surface regions. In addition, nanograins, dislocation substructures, and residual strain can influence the Cu surface work function through changes in crystallographic orientation, defect structure, and surface dipoles [31, 32, 33]. These effects provide a microstructural basis for the work function heterogeneity.

Microstructural refinement and defects generated by deformation are expected to increase the measured hardness of the region near the surface. We used nanoindentation [34] to quantify relative strengthening near the surface of graphene/Cu foils after laser shock. Because the comparison focuses on the processed surface layer, the analysis was restricted to shallow indentation depths. At shallow depth, surface topography can bias the apparent contact depth and increase measurement scatter. We sampled regions with both high and low surface relief. The high‐relief regions display broader dispersion and a higher fraction of outliers (Figure S4). The analysis below therefore focuses on regions with low surface relief to compare the strengthening trends before and after shock. After laser shock, the hardness *H* increases for both monolayer and bilayer graphene samples (Figure 2i). For monolayer graphene, *H* increases from 0.812 ± 0.025 GPa (A) to 1.636 ± 0.049 GPa (B). For bilayer graphene, *H* increases from 0.737 ± 0.027 GPa (C) to 1.400 ± 0.095 GPa (D). Representative load–displacement curves are shown in Figure 2j. Under the same peak load, the maximum indentation depth, *h*_max_, decreases systematically after shock, from 743 nm in A to 533 nm in B and from 763 nm in C to 592 nm in D. Together, the decrease in *h*_max_ and the increase in *H* are consistent with enhanced resistance to plastic flow in the processed region near the surface. This strengthening is attributed to plastic deformation at high strain rates during laser shock, which increases the density of deformation defects and introduces compressive residual stress into the processed layer, thereby increasing the flow stress and hardness [28, 29].

Raman spectroscopy reveals changes related to defects and disorder in graphene on Cu foils after laser shock and helps track the coupled evolution of strain and charge doping. Figure 3a presents Raman spectra of pristine CVD graphene/Cu foils (A and C) and the corresponding laser‐shocked foils (B and D). In pristine A and C, which correspond to monolayer and bilayer graphene, respectively, the D band activated by defects is barely detectable, and the 2D band remains relatively narrow. By contrast, B and D after laser shock exhibit distinct D and D′ bands together with a broadened 2D band [35]. Statistical analysis of the fitted Raman parameters is summarized in Figure 3b. *I*_2D_/*I*_G_ decreases overall in samples B and D relative to the pristine controls A and C, indicating a departure from graphene with low disorder. Because *I*_2D_/*I*_G_ is also sensitive to charge doping and changes in electronic scattering, this trend is interpreted together with *I*_D_/*I*_D′_ and the full width at half maximum (FWHM) of the 2D band. For B and D, *I*_D_/*I*_D′_ mainly falls in the range of 4–8, which commonly suggests a mixed defect population with major contributions from vacancies and boundary defects rather than predominantly sp^3^ functionalization [36]. In parallel, FWHM(2D) increases markedly after processing compared with the pristine controls. This broadening suggests increased heterogeneity below the Raman spot size in local lattice deformation and the electronic environment [37]. As illustrated in Figure 3c, the Raman spot typically spans multiple subregions of a protrusion, including the top, slope, root, and neighboring flat area, and therefore averages over regions with different local strain states and different degrees of interfacial coupling.

**FIGURE 3:**
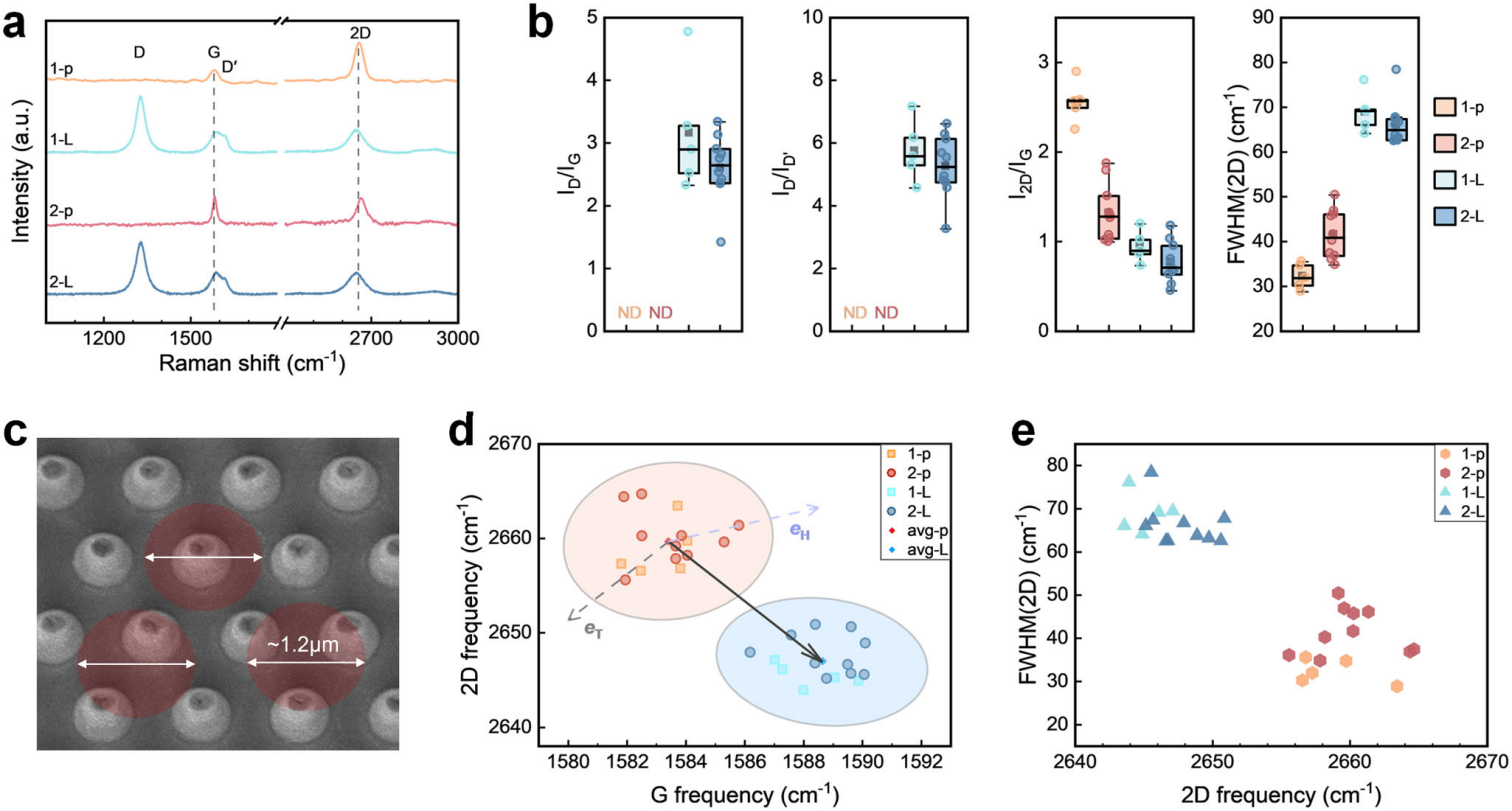
Raman evidence for defect generation and coupled strain–doping evolution after laser shock. (a) Representative Raman spectra of graphene/Cu foils before and after laser shock, measured with 633 nm excitation. Samples after laser shock exhibit clear D and D′ bands together with a broadened 2D band. (b) Statistical comparison of Raman fitting parameters, including *I*_D_/*I*_G_, *I*_D_/*I*_D′_, *I*_2D_/*I*_G_ and full width at half maximum of 2D band, FWHM(2D), extracted from the D, G, D′ and 2D bands. The D band was not detected (ND) in pristine 1‐p and 2‐p samples. (c) SEM image of a representative protrusion array with overlaid circles indicating the approximate Raman laser spot size. The panel illustrates that a single Raman acquisition can sample multiple local regions of the protrusion array, including protrusion tops, sidewalls or roots, and neighboring flat regions. (d) Correlation plot of *ω*_2D_ versus *ω*_G_. After laser shock, the data cloud shifts toward higher *ω*_G_ and lower *ω*_2D_. The shaded ellipses denote 95% confidence ellipses, and the dashed guide lines indicate reference directions associated with strain and doping effects. (e) Plot of FWHM(2D) versus *ω*_2D_. Samples after laser shock have lower *ω*_2D_ values and broader 2D linewidths than pristine samples. For 1‐p and 1‐L, n = 5; for 2‐p and 2‐L, n = 10. In (d), data from the pristine groups and groups after laser shock are summarized by group centroids, with n = 15 each.

Figure 3d summarizes the positions of the G and 2D peaks. Before shock, data from A and C mainly fall within 1581.8–1585.8 cm^−1^ for *ω*_G_ and 2655.6–2664.7 cm^−1^ for *ω*_2D_; after shock, data from B and D shift to 1586.2–1590.1 cm^−1^ for *ω*_G_ and 2643.9–2650.9 cm^−1^ for *ω*_2D_. The centroid of the data cloud shifts from (1583.41, 2659.69) cm^−1^ to (1588.64, 2647.01) cm^−1^, corresponding to a pronounced redshift of the 2D peak together with a blueshift of the G peak after laser processing. We further analyzed the *ω*_G_–*ω*_2D_ correlation by decomposing the shift vector into reference components associated with charge doping and strain, following the established correlation method [38, 39]. Under 633 nm excitation, the commonly used reference line slopes in the *ω*_G_–*ω*_2D_ plot are 0.7 for the doping axis and 2.2–2.5 for the strain axis [40] (2.45 in the figure). The observed trends support increased tensile strain together with a tendency toward hole doping (Note S2). Figure 3e plots FWHM(2D) against *ω*_2D_. In B and D, the redshift of the 2D peak is accompanied by an increase in FWHM(2D), which supports the presence of tensile strain together with stronger spatial heterogeneity in local strain and interfacial coupling. This finding supports the conclusion that laser shock does not generate a spatially uniform tensile state, in agreement with the analysis in Figure 3d. In addition, the increase in defect density after shock can also contribute to the broadening of the 2D peak linewidth by shortening phonon lifetimes. Taken together, the coupled evolution of *ω*_2D_ and FWHM(2D) reflects the combined effects of heterogeneity driven by morphology and defect generation under laser shock. After laser shock, graphene develops a more heterogeneous electronic landscape that contributes to local barrier variation relevant to field emission hot spots.

XPS shows that laser shock increases the chemical complexity of the region near the surface of graphene/Cu foils (Table S1). In the C 1s spectra of Figure 4a, the dominant contribution arises from carbon with sp^2^ bonding (C═C), together with components related to defects and functionalization, including sp^3^ C─C, C─O, and C═O/O─C═O. Relative to the unshocked samples A and C, the shocked samples B and D exhibit a reduced sp^2^ fraction and increased sp^3^ and oxygen‐containing components. This trend points to increased structural disorder and oxidation of graphene. The clear D and D′ bands observed in the Raman spectra of B and D support the same interpretation. After laser shock, the surface carbon environment evolves from carbon with predominantly sp^2^ bonding to a more chemically heterogeneous mixture that includes defect sites and oxygenated groups. This change is compatible with interfacial friction during plastic deformation of graphene/Cu and with local exposure of graphene edges and defect sites generated during shock deformation. The O 1s spectra contain increased contributions from oxygenated carbon species, including C─O and C═O/O─C═O, while Cu─O‐related components are also observed. These features may reflect the formation of a thin local oxide layer where Cu becomes exposed at graphene defect sites. In the Cu 2p spectra, metallic Cu remains the dominant component in all samples, and no clear Cu(II) shake‐up satellite is observed. To better distinguish Cu_2_O (Cu(I)) from metallic Cu, we additionally collected Cu LMM (Cu L_3_M_4,5_M_4,5_) Auger spectra. The Cu LMM peak positions change little for most samples, whereas one monolayer graphene sample after laser shock shows a pronounced peak shift together with an enhanced feature associated with Cu_2_O (Figure 4b). This result indicates that Cu_2_O formation is not uniform across the shocked samples but is locally promoted under specific processing and structural conditions. After laser shock, the oxygen content near the surface increases in B and D, with D showing the highest oxygen content among all samples (Figure 4c,d). Regional SEM–EDS comparisons also show relative oxygen enrichment after laser shock (Figure S5). The appearance of Cu_2_O further implies that laser shock promotes an oxygen‐containing environment near the surface, potentially involving surface oxides or hydroxyl species and oxygen intercalation at the graphene/Cu interface. Oxygen intercalation has been reported to decouple graphene from Cu and to modify charge transfer and energy level alignment [41, 42]. The work function and electron emission performance of graphene/Cu cathodes should therefore be interpreted in conjunction with the local microstructure, surface chemistry, and graphene/Cu interfacial coupling.

**FIGURE 4:**
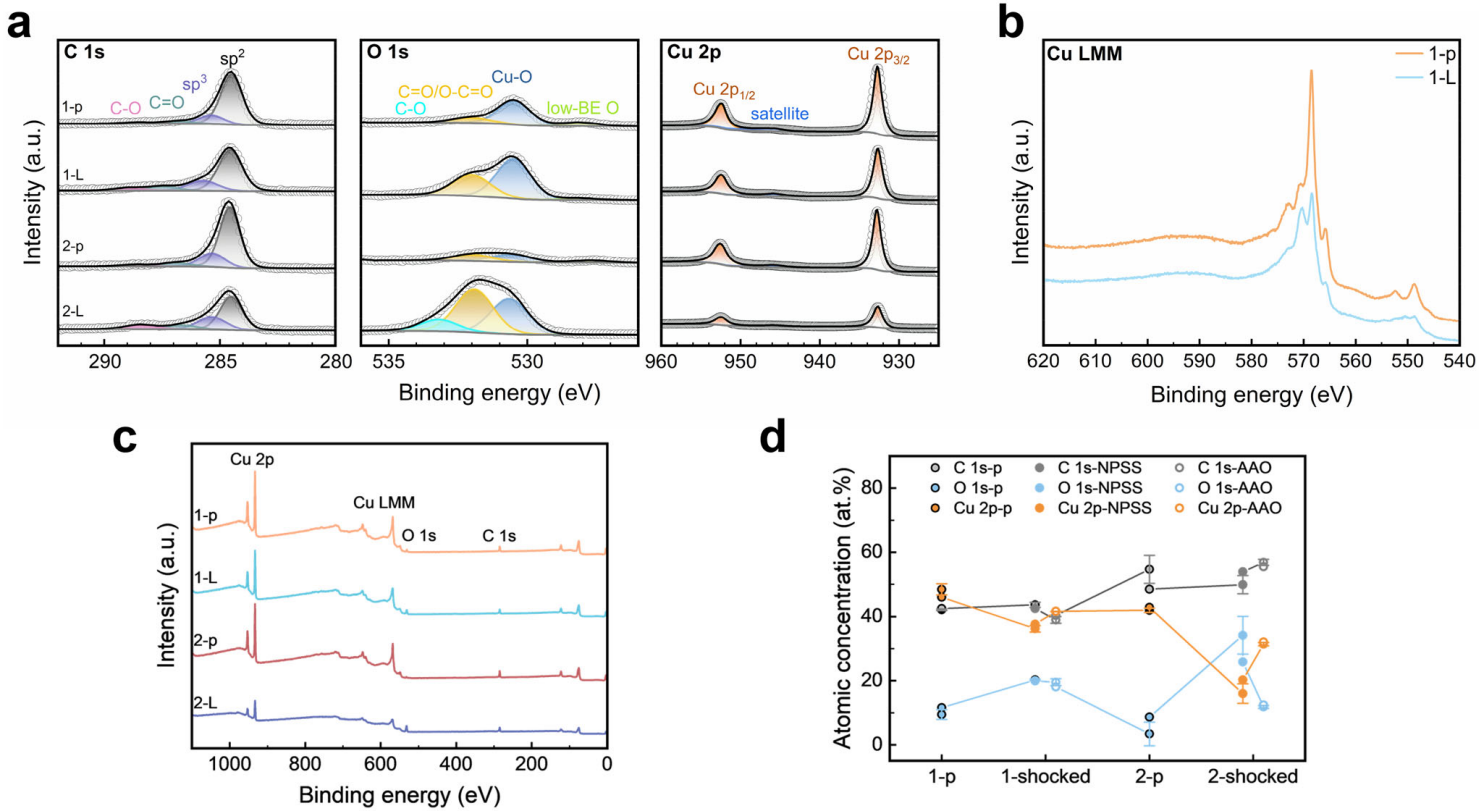
Surface chemical states after laser shock. (a) High‐resolution XPS C 1s, O 1s and Cu 2p spectra of graphene/Cu foils before and after laser shock, used to assess carbon bonding, oxygenated components and Cu chemical states. In (a), low‐BE O denotes the low‐binding‐energy oxygen component. (b) Cu LMM (Cu L_3_M_4,5_M_4,5_) Auger spectra from selected monolayer samples, used to compare Cu(0)‐ and Cu(I)‐related spectral features. (c) XPS survey spectra of 1‐p, 1‐L, 2‐p and 2‐L. (d) Relative atomic concentrations of C, O and Cu derived from XPS survey spectra for pristine samples and samples laser‐shocked using NPSS or AAO templates. Symbols and error bars denote means ± s.d. (n = 2 independent samples). In (d), shocked samples are grouped by graphene layer number, and the specific template is indicated in the legends. NPSS denotes nano‐patterned sapphire substrate, and AAO denotes anodic aluminum oxide.

### 2.3 Work Function Modulation by Laser Shock

Spatially resolved KPFM was used to examine local work function modulation within the micro‐ and nanoscale structures formed by laser shock (Figure 5). In graphene/Cu, the work function depends on the graphene overlayer, interfacial charge transfer, and interfacial dipoles, which are sensitive to graphene/Cu spacing, bonding configuration, local chemistry, and Cu surface structure [17, 18, 43].

**FIGURE 5:**
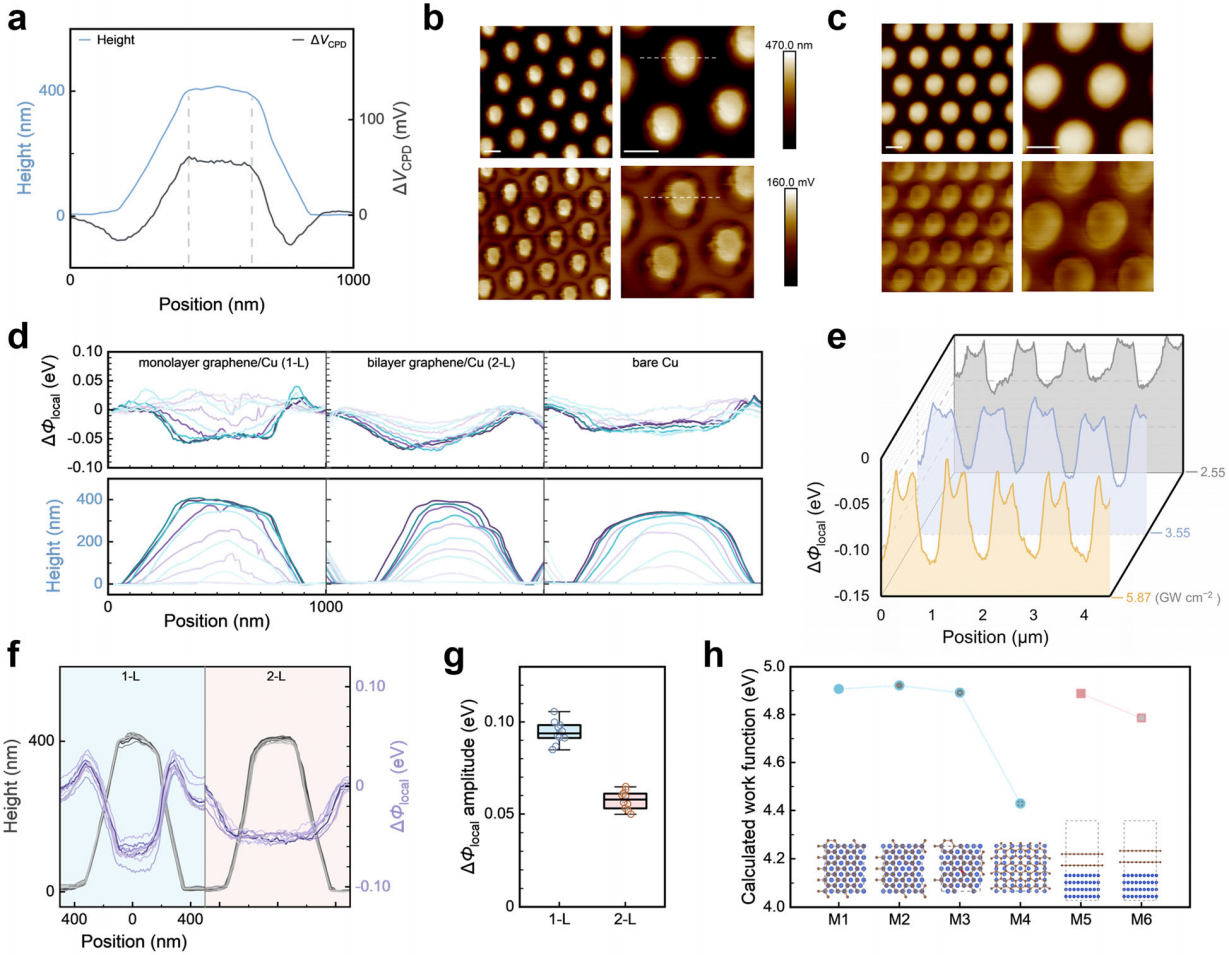
Local work function modulation resolved by KPFM and supported by DFT calculations. (a) Height and relative contact potential difference (Δ*V*_CPD_) profiles across a graphene/Cu protrusion after laser shock, extracted along the dashed line in b, showing surface potential modulation coupled to protrusion geometry. (b) AFM topography maps and paired Δ*V*_CPD_ maps of monolayer graphene/Cu protrusion arrays after laser shock. (c) AFM topography maps and paired Δ*V*_CPD_ maps of bare Cu protrusion arrays after laser shock. The same height and Δ*V*_CPD_ color scales are used in (b) and (c). Scale bars, 500 nm. (d) Relative local work function (Δ*Φ*_local_) and height profiles extracted across monolayer graphene/Cu, bilayer graphene/Cu, and bare Cu protrusions after laser shock. (e) Representative Δ*Φ*_local_ profiles of monolayer graphene/Cu protrusion arrays processed at laser power densities of 2.55, 3.55, and 5.87 GW cm^−2^. (f) Height and Δ*Φ*_local_ profiles extracted along cross sections through the maximum height of multiple protrusions in monolayer and bilayer graphene/Cu samples (n = 10 individual protrusions collected from multiple scan regions). (g) Statistical comparison of the local work function modulation amplitude extracted from the profiles in (f). For each profile, the amplitude is calculated as the difference between the maximum and minimum Δ*Φ*_local_. (h) Calculated work functions for simplified DFT models, including monolayer graphene/Cu(111) (M1), monolayer graphene/Cu(111) with a graphene vacancy (M2), monolayer graphene/Cu(111) with a C–O bridge configuration on graphene (M3), monolayer graphene on stepped Cu(211) (M4), bilayer graphene/Cu(111) (M5) and bilayer graphene/Cu(111) with increased graphene/Cu spacing (M6). Brown, blue and red spheres represent C, Cu and O atoms, respectively. Enlarged views of M1–M6 are provided in Figure S6. Δ*Φ*_local_ was calculated from the relative CPD contrast using Δ*Φ*_local_ = −*e* · Δ*V*_CPD_, where *e* is the elementary charge.

To resolve surface potential variation across different regions of the protrusions formed by laser shock, we performed KPFM mapping of the contact potential difference (CPD, denoted as *V*_CPD_) on graphene/Cu. A bias voltage was applied to the probe tip, and the sign convention used here is *V*_CPD_ = *V*_sample_ − *V*_tip_. Using the same probe under matched measurement conditions, spatial variations in *V*_CPD_ within a given map are mainly interpreted as contrast arising from the sample. Accordingly, the relative local work function change, Δ*Φ*_local_, is discussed as Δ*Φ*_local_ = −*e* · Δ*V*_CPD_, where *e* is the elementary charge [44, 45, 46]. As shown in Figure 5b, the monolayer graphene sample B after laser shock exhibits localized periodic modulation in *V*_CPD_ that follows the protrusion geometry. Figure 5a plots the topography and *V*_CPD_ along a line profile through the maximum cross section of a representative protrusion. The modulation amplitude across a single protrusion reaches 95.5 mV in *V*_CPD_, corresponding to a local work function contrast of 0.095 eV. Moving from the flat substrate region toward the root region, *V*_CPD_ decreases sharply and reaches a minimum near the root region. Along the sidewall, *V*_CPD_ then increases gradually with height. The highest *V*_CPD_, which represents the lowest local work function within the same map, typically appears near the apex or outer rim of the protrusion. This sequence from the root to the sidewall and then to the top region, namely high barrier at the root region, intermediate barrier along the sidewall region, and low barrier at the top region, indicates that laser shock does not impose a uniform work function shift across the entire protrusion. Instead, laser shock creates work function modulation that depends on geometry on the submicrometer scale.

The spatial contrast in local work function agrees with the strongly nonuniform deformation within the protrusion, as discussed in the TEM section. The root region experiences severe friction and shear during forming, and graphene continuity is locally disrupted. The intense plastic deformation can also reconstruct the Cu substrate, increase defect density, reduce grain size, and generate a complex orientation distribution. On this basis, the higher *Φ*_local_ near the root can be attributed to a combination of factors, including graphene defects and edge states together with local oxygen functionalization [47], the sensitivity of the Cu work function to crystallographic orientation and strain, and changes in interfacial charge transfer at the graphene/Cu interface.

To separately evaluate the contributions of Cu and graphene layers to work function modulation, we performed KPFM on the bilayer graphene/Cu sample D and on a bare Cu foil control, and compared their height and local work function profiles with those of monolayer graphene/Cu (Figure 5c,d). For all samples, the local maximum in *Φ*_local_ is typically located in the root region or the nearby flat substrate region, whereas regions with lower *Φ*_local_ are mainly distributed across the sidewall and top regions. Compared with monolayer graphene/Cu (B), bilayer graphene/Cu (D) retains significant modulation, but with a different spatial distribution. In sample D, although Δ*Φ*_local_ along the maximum cross section still reaches approximately 0.07 eV, the low *Φ*_local_ region covers a larger fraction of the protrusion surface, whereas the high *Φ*_local_ band near the root region is less pronounced. These differences demonstrate that the local work function landscape is layer dependent, with graphene layer number altering the relative contributions of graphene disorder, surface chemistry, Cu microstructure, and interfacial charge transfer. Bilayer graphene differs from monolayer graphene in electrostatic screening and interlayer coupling, and metal substrates can further modify its electronic structure through charge transfer that depends on layer number and changes in graphene–metal spacing [48, 49]. In sample D, the surface and interfacial environment containing oxygen, as inferred from XPS, may contribute to partial decoupling at the graphene/Cu interface. This effect, together with interlayer sliding and bilayer screening, can reduce the sensitivity of *Φ*_local_ to deformation near the root, making the high *Φ*_local_ band less pronounced than in monolayer graphene/Cu. Because the work function of Cu is sensitive to crystal orientation, dislocations, and strain, the bare Cu control also exhibits *Φ*_local_ modulation. However, for comparable protrusion heights, the modulation amplitude of *Φ*_local_ is substantially smaller than that observed for graphene/Cu, indicating that graphene makes a substantial contribution. This difference suggests that graphene amplifies the work function response beyond that produced by Cu deformation alone, reflecting the combined influence of the graphene overlayer and graphene/Cu coupling that is sensitive to local structure. Together, these factors enhance the sensitivity of surface dipoles and interfacial charge transfer to local structure and strain, thereby amplifying work function modulation.

In addition, the amplitude of work function modulation can be tuned by the laser shock parameters. In monolayer graphene/Cu, the protrusion height increases with laser power density across 2.55, 3.55, and 5.87 GW cm^−2^. Over this series, the representative local work function modulation amplitude increases from approximately 0.03 to 0.12 eV (Figure 5e). Statistical analysis of multiple protrusions formed under the same conditions (Figure 5f,g) indicates similar morphology and spatially regionalized *Φ*_local_ distributions, but the modulation amplitude in monolayer graphene/Cu is consistently greater than that in bilayer graphene/Cu.

To examine how representative structural and interfacial changes affect the work function, we constructed simplified DFT models guided by the experimental observations (Figure 5h and Table S2). For monolayer graphene/Cu, models M1–M4 represent the effects of graphene defects, oxygenated functionality, and Cu surface configuration. Relative to the baseline graphene/Cu(111) model (M1), introducing a graphene single vacancy defect (M2) slightly increases the work function, whereas a C─O bridge configuration on graphene (M3) and a stepped Cu(211) surface (M4) decrease the work function. To represent differences between bilayer and monolayer graphene/Cu, we simplified the effects of possible interlayer sliding and oxygen intercalation into effective interfacial decoupling and constructed models M5 and M6. Increasing the graphene/Cu spacing in M6 decreases the work function relative to M5. Together with the experimental observations, these calculations rationalize how local work function modulation arises from the combined and sometimes competing effects of graphene defects, surface chemistry, Cu surface configuration, and graphene/Cu interfacial coupling. Accordingly, materials with tailored work function characteristics can be designed for different applications. For field emission cathodes, performance is directly related to surface work function and, in practice, mainly to local hot spots. The top and rim regions combine high local curvature with lower *Φ*_local_ and provide a physical basis for field emission dominated by hot spots.

### 2.4 Geometry and Work Function Coupling in Field Emission

Field emission characteristics of graphene/Cu cathodes with a micro‐ and nanostructured surface were measured in an ultrahigh vacuum environment (Figure 6a). The macroscopic electric field is denoted as *E* = *V*/*d*, and the macroscopic current density is denoted as *J* = *I*/*A*. Within the field range shown in Figure 6b, no field emission current was detected in A and C. In contrast, after laser shock, current signals were detected in B and D, with a turn‐on field down to 4.9 V µm^−1^ and a current density reaching 2.2 mA cm^−2^, demonstrating a substantial enhancement in field emission performance. Four independently fabricated bilayer graphene/Cu cathodes prepared under the same processing conditions gave *E*_on_ = 7.35 ± 0.16 V µm^−1^ (Figure S7). Under the same laser shock conditions, the bilayer graphene/Cu cathode has higher *E*_on_ and *E*_th_. This contrast is consistent with the different local work function distributions resolved by KPFM and indicates that graphene layer number affects the field emission response, providing an additional tuning parameter. The Fowler–Nordheim (FN) plot (Figure 6b) contains an approximately linear region, suggesting that emission is dominated by FN tunneling. The apparent field enhancement factor was defined as *β*_app_ = −*bΦ*_ref_^3/2^/*S*_FN_ using the elementary FN relation [8], where *S*_FN_ is the slope of a linear fit to ln(*J*/*E* ^2^) versus 1/*E* and *b* is the second FN constant. A fit for a separately optimized monolayer graphene/Cu cathode gave *β*_app_ ≈ 5 × 10^3^ (Figure S8). To investigate where the specified geometry concentrates the applied electric field, we used cathode protrusion geometries extracted from SEM and AFM observations for finite element electrostatic simulations (Figure 6c and Figure S9). The simulated field distribution reveals strong local field enhancement near the protrusion top region, where KPFM resolves lower *Φ*_local_. A stronger local field narrows the tunneling barrier, whereas a lower *Φ*_local_ reduces its zero‐field height. This spatial correspondence provides a physical basis for preferential emission from these regions. The measured current sums local emission over the active emitting area, while the local emission current density depends nonlinearly on the local field and barrier [50]. Because laser shock changes geometry and local work function simultaneously, the present measurements do not separately quantify their contributions to the emission enhancement. To clarify the role of graphene, bare Cu cathodes were tested after processing with the same templates and laser conditions (Figure S10). Pristine Cu and processed Cu with shallow protrusions showed no detectable emission below 15 V µm^−1^. At the higher laser power density, bare Cu produced measurable emission but exhibited lower current densities and larger current variations during voltage sweeps than graphene/Cu. These controls, together with the larger relative local work function contrast in graphene/Cu at comparable protrusion heights (Figure 5d), support an electronic contribution from graphene in addition to geometric field concentration. Graphene/Cu coupling can modify the interfacial potential and electron transmission into vacuum. Graphene coverage can also reduce residual gas adsorption and protect Cu during air exposure [51], which may contribute to the smaller current variations during voltage sweeps.

**FIGURE 6:**
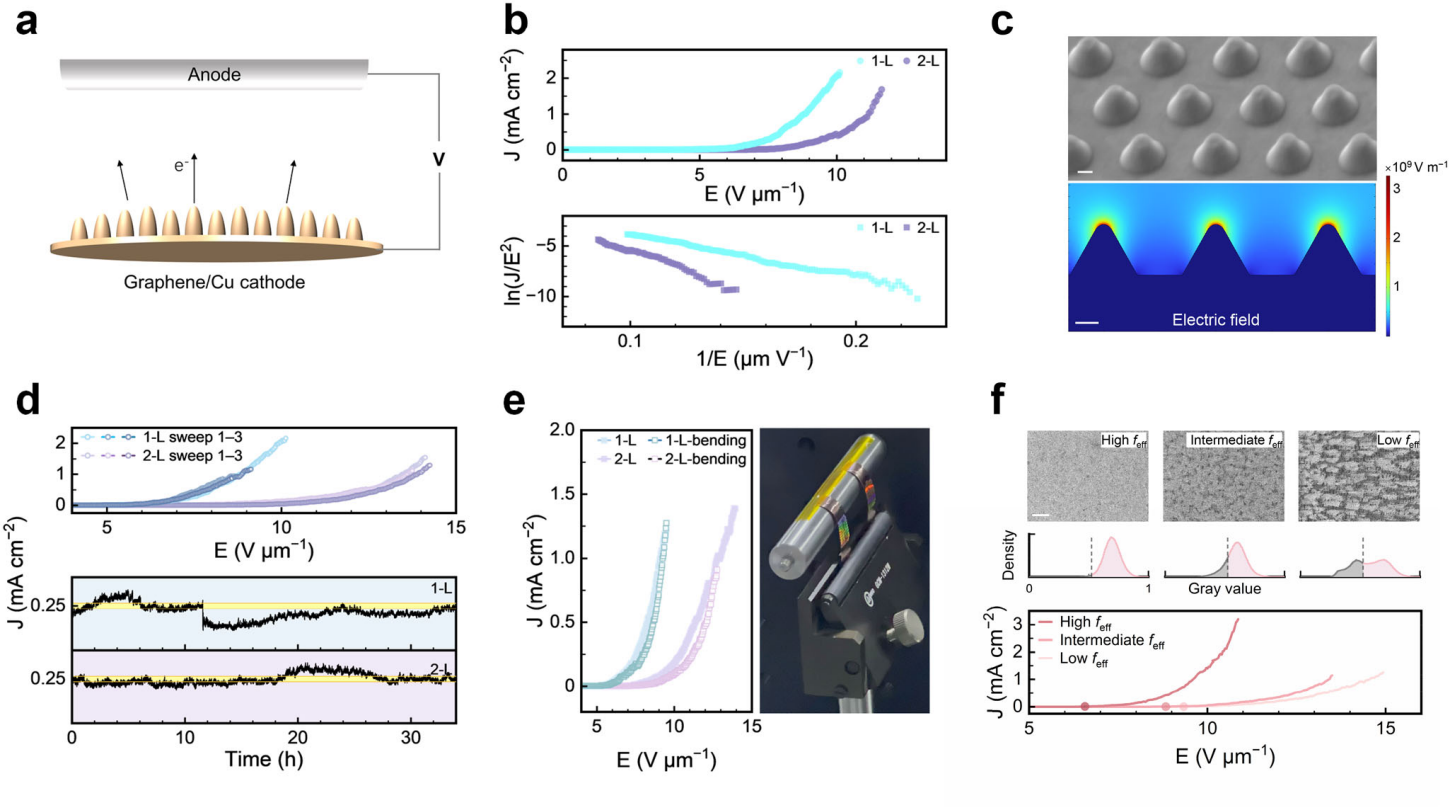
Field emission performance, stability and bending durability of laser‐shocked graphene/Cu cathodes. (a) Schematic illustration of the field emission measurement configuration, with a laser‐shocked graphene/Cu cathode facing a planar stainless‐steel anode. (b) Field emission current density–electric field (*J*–*E*) curves and corresponding Fowler–Nordheim plots for monolayer and bilayer graphene/Cu cathodes after laser shock. (c) SEM image of a protrusion array and electrostatic simulation of the local electric field near the protrusion apex. The color scale indicates the electric field magnitude in units of 10^9^ V m^−1^. Scale bars, 200 nm. (d) Repeated *J*–*E* sweeps and continuous emission stability traces for monolayer and bilayer graphene/Cu cathodes after laser shock. Stability tests were performed under constant electric fields selected to give an initial current density of 0.25 mA cm^−2^. Yellow bands mark 95–105% of the initial current density. (e) Field emission characteristics before and after 3000 bending cycles for monolayer and bilayer graphene/Cu cathodes after laser shock, together with the bending test configuration. (f) Representative InLens SEM images, normalized gray‐value histograms and *J*–*E* curves for laser‐shocked cathodes with high, intermediate and low effective treated area fractions, *f*_eff_. The dashed lines in the histograms mark the threshold used to classify the effective treated area. Scale bars, 50 µm. The turn‐on field *E*_on_ is defined at *J* = 10 µA cm^−2^ and the threshold field *E*_th_ is defined at *J* = 1 mA cm^−2^. 1‐L and 2‐L denote monolayer and bilayer graphene/Cu cathodes after laser shock, respectively.

We further evaluated the continuous emission stability of the cathodes. Under a constant electric field corresponding to an initial current density of 0.25 mA cm^−2^, both monolayer and bilayer graphene/Cu cathodes retained more than 95% of their initial emission current over 32 h, demonstrating stable operation (Figure 6d). Current fluctuations occurred during operation and were more pronounced for the monolayer graphene/Cu cathode, but the emission current gradually returned close to the baseline. Repeated *J*–*E* measurements on the same tested cathodes after the continuous stability test did not lead to a substantial increase in the turn‐on field or threshold field, and the emission characteristics showed only minor variations between cycles. During the continuous emission, adsorption and desorption kinetics can perturb emission from individual nanoscale sites [52], and thermal effects can further destabilize active sites. The presence of numerous emission hot spots may allow emission to redistribute among active sites, so that deactivation of some sites is offset by activation of others, helping to maintain the overall cathode output. At high current, gas release from the polymer‐containing adhesive and restricted heat removal through the mounting contact are possible contributors to instability alongside emission‐induced changes at active sites. Taking advantage of the flexibility of the Cu substrate, we also performed repeated bending tests to examine field emission performance under repeated mechanical deformation. After 3000 bending cycles, the turn‐on field of both monolayer and bilayer graphene/Cu cathodes increased only slightly, whereas the maximum current density showed no monotonic degradation (Figure 6e). Collectively, laser shock processing enhances field emission while preserving emission stability and bending durability in flexible graphene/Cu cathodes.

Graphene/Cu cathodes with different treated areas and nanostructures further demonstrate that field emission can be tuned through the activation of candidate hot spots. For closely packed nanostructured cathodes, emission performance improves relative to the untreated cathode, but the maximum current density remains limited (Figure S11). This behavior can be attributed to electrostatic screening caused by small emitter spacing, which suppresses the local field enhancement of individual emitters [53, 54]. For cathodes with a hierarchical surface structure, we used InLens SEM images to quantify the effective treated area fraction, *f*_eff_, as a relative metric for the macroscopic coverage of regions modified by laser shock (Note S3 and Figure S12). A larger *f*_eff_ leads to a lower *E*_on_ and a higher *J*_max_ (Figure 6f). This trend supports the interpretation that a larger treated area increases the population of candidate hot spots by providing more edges, defects, and sharp geometric features. However, as suggested by the closely packed nanostructures, an excessively high site density can also strengthen field screening. Practical optimization thus requires a balance between hot spot density and screening. As a scalable process, laser shock provides a simple route for tuning field emission performance over a relatively wide range.

## 3 Conclusions

The central implication of this study is that forming at high strain rates can define both the geometry of graphene on metal and the spatial distribution of the local emission barrier. In graphene/Cu foils, laser shock microforming creates protrusion arrays and introduces heterogeneity in graphene disorder and strain, Cu microstructure, surface chemical state and graphene/Cu interfacial coupling, producing local work function contrasts reaching 0.12 eV within individual monolayer graphene/Cu protrusions. These structural, chemical and interfacial variations are coupled to surface geometry: regions with lower *Φ*_local_ are located near protrusion tops and rims, where simulations indicate strong electric field concentration. This spatial correspondence supports the joint influence of geometric field enhancement and reduced local barriers on field emission. The cathodes exhibit lower turn‐on fields, higher emission current densities, stable operation over 32 h at 0.25 mA cm^−2^ and durability after 3000 bending cycles. Comparisons across graphene layer number and morphology further show that this response is tunable through the spatial weighting of regions with different *Φ*_local_, the density of candidate emission sites and the degree of electrostatic screening. By converting heterogeneity generated by laser shock into a variable for emission design, laser shock microforming offers a route for manufacturing flexible graphene/metal cold cathodes over large areas without transfer. The relative contributions of local work function modulation and electrostatic field concentration to the emission enhancement remain to be quantified separately. Further optimization can focus on preserving this geometry–barrier alignment during operation at higher current densities by improving cathode heat removal, stabilizing the graphene/Cu interface, and limiting adsorption, oxidation, and thermally driven changes at active sites.

## 4 Methods

### 4.1 Graphene/Cu Foil Preparation and Laser Shock Imprinting

For direct comparison, we used commercially available Cu foils (25 µm thick; ACS Materials) with monolayer or bilayer graphene. Laser shock processing was carried out using an Nd:YAG nanosecond pulsed laser (Nimma, Beamtech Laser Technology, 1064 nm, 9 ns). A 25‐µm‐thick Al foil with a graphite coating was used as the absorption layer, and a 3‐mm‐thick K9 glass plate was used as the transparent confinement layer. The stack was mechanically clamped to maintain intimate contact among all layers. Patterning was performed using a nano‐patterned sapphire substrate (NPSS) template with a period of 1 µm and inverted conical holes of approximately 700 nm in diameter, anodic aluminum oxide (AAO) templates with nominal feature sizes of 125 and 450 nm, and nanodiamond grinding discs (3M, 661X Diamond lapping film). Unless otherwise stated, a representative processing condition used a laser spot diameter of 0.88 mm, a pulse energy of 260 mJ, and a scanning step of 0.75 mm. Patterning over a large area was achieved by scanning the laser spot using a motion stage.

### 4.2 Microstructural Characterization and Mechanical Properties

Surface morphologies were examined by SEM (Zeiss Gemini 500) and AFM (Bruker Dimension Icon). Focused ion beam (FIB) lamellae were prepared through the maximum cross section of individual protrusions. To protect the graphene layer during FIB preparation, an amorphous carbon protective layer was deposited, followed by Pt deposition at low current, and the lamellae were thinned to approximately 40 nm. Transmission electron microscopy (TEM) and scanning transmission electron microscopy (STEM) images were acquired on an FEI Talos F200X operated at 200 kV. Higher resolution integrated differential phase contrast STEM images were acquired on an aberration‐corrected Thermo Fisher Spectra 300 operated at 60 or 300 kV. The visibility of carbon atoms was enhanced by adjusting the imaging conditions and specimen tilt. Nanoindentation was performed using load control on a Keysight Nano Indenter G200: linear loading to 10 mN over 15 s, a 10 s hold at peak load, unloading to 1 mN over 15 s, a 75 s hold at low load to assess thermal drift, and final unloading. Raman spectra were acquired on a HORIBA LabRAM HR Evolution using 633 nm excitation to reduce interference from the Cu background, with a 50× objective, a laser power of 0.77 mW, an integration time of 30 s per spectrum, and two accumulations. Lorentzian peak fitting was used to extract the D, G, D′, and 2D intensities and FWHM(2D).

### 4.3 Chemical State Near the Surface

XPS was performed using a monochromatic Al Kα X‐ray source (PHI GENESIS 900; spot size 200 µm), and binding energy was calibrated based on the Cu 2p peak position. The acquisition conditions and secondary electron cutoff assessment for ultraviolet photoelectron spectroscopy are provided in Figure S14.

### 4.4 Local Work Function Mapping and Simulation

KPFM measurements were carried out on a scanning probe microscope (Dimension Icon, Bruker), using a probe with a Cobalt‐Chromium conductive coating (MESP‐RC‐V2, Bruker). A lift height of 135 nm was selected to reduce interference from morphology and instability. The probe condition was checked using a freshly prepared Au reference surface during the KPFM measurement sequence (Figure S15). Maps acquired with the same probe were considered reliable when the CPD measured on the Au reference showed limited temporal drift during the corresponding measurement sequence. The flat substrate region within each graphene/Cu map was used as the local reference for relative CPD analysis.

The work function in the DFT simulations was calculated using VASP [55] as Φ  =  *V*_vacuum_−*E*_F_, where *V*_vacuum_ is the vacuum electrostatic potential extracted after planar averaging, and *E*_F_ is the Fermi level. The main calculation parameters [56, 57] were ENCUT = 500 eV, GGA = PBE, EDIFF = 1 × 10^−5^ eV, LDIPOL = True and IDIPOL = 3. Brillouin zone sampling employed a Γ‐centered 4 × 3 × 1 *k*‐point mesh. A force‐stopping criterion of 0.02 eV Å^−1^ was specified for structural relaxation. A vacuum thickness of 15 Å was used during initial model construction. The graphene/Cu separation is defined along the surface normal between the mean atomic plane of the lower graphene layer and that of the uppermost Cu layer. Compared to M5, M6 was constructed by increasing this separation by 1 Å. Simplified graphene/Cu models were constructed to isolate representative factors identified experimentally. The model definitions and calculated work functions are summarized in Table S2. Enlarged structural views and model dimensions are provided in Figure S6.

### 4.5 Field Emission Measurements and Electrostatic Simulations

Field emission measurements were performed at a base pressure of 10^−7^ Pa. The cathode was a graphene/Cu foil processed by laser shock (10 mm × 10 mm), and the anode was a planar stainless steel electrode. The cathode–anode gap *d* was kept constant during each measurement at a value of several hundred micrometers, such as 300 µm, and *A* denotes the geometric cathode area. Before measurements, the cathode was fixed to a rigid metal stage and then conditioned at low field to reduce nonideal effects such as gas adsorption. The bias voltage was increased in constant steps; each step was held for 3 s, and the reported current was taken as the average of three readings collected at 1 s intervals. During the bending experiment, the two ends of the graphene/Cu foil were fixed to a stationary support and a motorized translation stage for reciprocating cycles. Electrostatic simulations were performed in COMSOL Multiphysics 6.0 using cathode protrusion geometries, with local mesh refinement near the protrusion top. The applied bias and gap between the cathode and anode were set to 5 kV and 300 µm, respectively, matching the field emission measurement conditions.

## Author Contributions

**Ximei Xu** and **Ming Zhou** conceived the project. Ximei Xu developed the methodology, carried out the experiments, performed the formal analysis, curated the data, and wrote the original manuscript. **Haonan Tao** contributed to the methodology, simulation, visualization, and manuscript revision. **Wenming Li** and **Xuyin Li** assisted with the experiments and data analysis. **Jiacong Lin** assisted with the methodology and experimental setup. **Mingyang Yang** provided resources, contributed to project administration, and revised the manuscript. Ming Zhou contributed to the methodology, supervised the research, acquired funding, and revised the manuscript. All authors contributed to the discussion of the results and approved the final manuscript.

## Conflicts of Interest

The authors declare no conflicts of interest.

## Supporting information




**Supporting File**: advs78333‐sup‐0001‐SuppMat.pdf.

## Data Availability

The data that support the findings of this study are available from the corresponding author upon reasonable request.

## References

[1] J. S. Han , S. H. Lee , H. Go , S. J. Kim , J. H. Noh , and C. J. Lee , “High‐Performance Cold Cathode X‐Ray Tubes Using a Carbon Nanotube Field Electron Emitter,” ACS Nano 16, no. 7 (2022): 10231–10241, 10.1021/acsnano.2c02233. 35687140

[2] D. Xiao , H. Du , L. Sun , et al., “Boosting the Electron Beam Transmittance of Field Emission Cathode Using a Self‐Charging Gate,” Nature Communications 15, no. 1 (2024): 764, 10.1038/s41467-024-45142-0. PMC10817892 38278943

[3] S.‐K. Jerng , E. Hong , G. Lee , et al., “Integration of Vertical Graphene Onto a Tunnelling Cathode for Digital X‐Ray Imaging,” Advanced Science 11, no. 39 (2024): 2403721, 10.1002/advs.202403721. PMC11497061 39148365

[4] S. Kang , G. Qi , W. Wu , et al., “Cold Cathode Flat Panel X‐Ray Source for Talbot–Lau Grating Interferometer using Zinc Oxide Nanowire Field Emitter Arrays and Periodic Microstructured Anode,” ACS Applied Nano Materials 7, no. 22 (2024): 25439–25446, 10.1021/acsanm.4c04410.

[5] C. A. Spindt , “A Thin‐Film Field‐Emission Cathode,” Journal of Applied Physics 39, no. 7 (1968): 3504–3505, 10.1063/1.1656810.

[6] R. H. Fowler and L. Nordheim , “Electron Emission in Intense Electric Fields,” Proceedings of the Royal Society of London Series A, Containing Papers of a Mathematical and Physical Character 119 (1928): 173–181, 10.1098/rspa.1928.0091.

[7] M. Zubair , Y. S. Ang , and L. K. Ang , “Fractional Fowler–Nordheim Law for Field Emission From Rough Surface With Nonparabolic Energy Dispersion,” IEEE Transactions on Electron Devices 65, no. 6 (2018): 2089–2095, 10.1109/TED.2017.2786020.

[8] R. G. Forbes , A. Fischer , and M. S. Mousa , “Improved Approach to Fowler–Nordheim Plot Analysis,” Journal of Vacuum Science & Technology B, Nanotechnology and Microelectronics: Materials, Processing, Measurement, and Phenomena 31, no. 2 (2013): 02B103, 10.1116/1.4765080. PMC3634314 23847750

[9] C. H. Yoon , G. M. Seo , S. H. Yoon , and B. D. Kong , “Field Emission Control by Work Function Modulation in Graphene Edge Cathodes,” Journal of Applied Physics 136, no. 6 (2024): 064501, 10.1063/5.0215449.

[10] Y.‐J. Yu , Y. Zhao , S. Ryu , L. E. Brus , K. S. Kim , and P. Kim , “Tuning the Graphene Work Function by Electric Field Effect,” Nano Letters 9, no. 10 (2009): 3430–3434, 10.1021/nl901572a. 19719145

[11] W. A. de Heer , A. Châtelain , and D. Ugarte , “A Carbon Nanotube Field‐Emission Electron Source,” Science 270, no. 5239 (1995): 1179–1180, 10.1126/science.270.5239.1179.

[12] J.‐M. Bonard , H. Kind , T. Stöckli , and L.‐O. Nilsson , “Field Emission From Carbon Nanotubes: The First Five Years,” Solid‐State Electronics 45, no. 6 (2001): 893–914, 10.1016/S0038-1101(00)00213-6.

[13] X. Shao , A. Srinivasan , W. K. Ang , and A. Khursheed , “A High‐Brightness Large‐Diameter Graphene Coated Point Cathode Field Emission Electron Source,” Nature Communications 9, no. 1 (2018): 1288, 10.1038/s41467-018-03721-y. PMC5876327 29599466

[14] Z. Xiao , J. She , S. Deng , et al., “Field Electron Emission Characteristics and Physical Mechanism of Individual Single‐Layer Graphene,” ACS Nano 4, no. 11 (2010): 6332–6336, 10.1021/nn101719r. 20929240

[15] T. Hallam , M. T. Cole , W. I. Milne , and G. S. Duesberg , “Field Emission Characteristics of Contact Printed Graphene Fins,” Small 10, no. 1 (2014): 95–99, 10.1002/smll.201300552. 23913746

[16] S. Tang , Y. Zhang , P. Zhao , R. Zhan , J. Chen , and S. Deng , “Realizing the Large Current Field Emission Characteristics of Single Vertical Few‐Layer Graphene by Constructing a Lateral Graphite Heat Dissipation Interface,” Nanoscale 13, no. 10 (2021): 5234–5242, 10.1039/D0NR09231B. 33620362

[17] G. Giovannetti , P. A. Khomyakov , G. Brocks , V. M. Karpan , J. van den Brink , and P. J. Kelly , “Doping Graphene With Metal Contacts,” Physical Review Letters 101, no. 2 (2008): 026803, 10.1103/PhysRevLett.101.026803. 18764212

[18] P. A. Khomyakov , G. Giovannetti , P. C. Rusu , G. Brocks , J. van den Brink , and P. J. Kelly , “First‐Principles Study of the Interaction and Charge Transfer Between Graphene and Metals,” Physical Review B 79, no. 19 (2009): 195425, 10.1103/PhysRevB.79.195425.

[19] X. Li , W. Cai , J. An , et al., “Large‐Area Synthesis of High‐Quality and Uniform Graphene Films on Copper Foils,” Science 324, no. 5932 (2009): 1312–1314, 10.1126/science.1171245. 19423775

[20] P. R. Kidambi , B. C. Bayer , R. Blume , et al., “Observing Graphene Grow: Catalyst–Graphene Interactions During Scalable Graphene Growth on Polycrystalline Copper,” Nano Letters 13, no. 10 (2013): 4769–4778, 10.1021/nl4023572. PMC3883115 24041311

[21] Y. Zhao , Y. Song , Z. Hu , et al., “Large‐Area Transfer of Two‐Dimensional Materials Free of Cracks, Contamination and Wrinkles via Controllable Conformal Contact,” Nature Communications 13, no. 1 (2022): 4409, 10.1038/s41467-022-31887-z. PMC9338253 35906212

[22] G. Lupina , J. Kitzmann , I. Costina , et al., “Residual Metallic Contamination of Transferred Chemical Vapor Deposited Graphene,” ACS Nano 9, no. 5 (2015): 4776–4785, 10.1021/acsnano.5b01261. 25853630

[23] C. Deng , W. Lin , G. Agnus , et al., “Reversible Charge‐Transfer Doping in Graphene due to Reaction With Polymer Residues,” The Journal of Physical Chemistry C 118, no. 25 (2014): 13890–13897, 10.1021/jp501077t.

[24] R. Fabbro , J. Fournier , P. Ballard , D. Devaux , and J. Virmont , “Physical Study of Laser‐Produced Plasma in Confined Geometry,” Journal of Applied Physics 68, no. 2 (1990): 775–784, 10.1063/1.346783.

[25] H. Gao , Y. Hu , Y. Xuan , et al., “Large‐Scale Nanoshaping of Ultrasmooth 3D Crystalline Metallic Structures,” Science 346, no. 6215 (2014): 1352–1356, 10.1126/science.1260139. 25504717

[26] D. Goswami , J. C. Munera , A. Pal , B. Sadri , C. L. P. G. Scarpetti , and R. V. Martinez , “Roll‐to‐Roll Nanoforming of Metals Using Laser‐Induced Superplasticity,” Nano Letters 18, no. 6 (2018): 3616–3622, 10.1021/acs.nanolett.8b00714. 29775318

[27] M. Motlag , P. Kumar , K. Y. Hu , et al., “Asymmetric 3D Elastic–Plastic Strain‐Modulated Electron Energy Structure in Monolayer Graphene by Laser Shocking,” Advanced Materials 31, no. 19 (2019): 1900597, 10.1002/adma.201900597. 30924972

[28] M. A. Meyers , F. Gregori , B. K. Kad , et al., “Laser‐Induced Shock Compression of Monocrystalline Copper: Characterization and Analysis,” Acta Materialia 51, no. 5 (2003): 1211–1228, 10.1016/S1359-6454(02)00420-2.

[29] M. S. Schneider , B. Kad , D. H. Kalantar , et al., “Laser Shock Compression of Copper and Copper–Aluminum Alloys,” International Journal of Impact Engineering 32, no. 1‐4 (2005): 473–507, 10.1016/j.ijimpeng.2005.05.010.

[30] J. W. Christian and S. Mahajan , “Deformation Twinning,” Progress in Materials Science 39, no. 1‐2 (1995): 1–157, 10.1016/0079-6425(94)00007-7.

[31] P. O. Gartland , S. Berge , and B. J. Slagsvold , “Photoelectric Work Function of a Copper Single Crystal for the (100), (110), (111), and (112) Faces,” Physical Review Letters 28, no. 12 (1972): 738–739, 10.1103/physrevlett.28.738.

[32] S. Tao and D. Y. Li , “Nanocrystallization Effect on the Surface Electron Work Function of Copper and Its Corrosion Behaviour,” Philosophical Magazine Letters 88, no. 2 (2008): 137–144, 10.1080/09500830701771939.

[33] W. Li and D. Y. Li , “Variations of Work Function and Corrosion Behaviors of Deformed Copper Surfaces,” Applied Surface Science 240, no. 1‐4 (2005): 388–395, 10.1016/j.apsusc.2004.07.017.

[34] W. C. Oliver and G. M. Pharr , “An Improved Technique for Determining Hardness and Elastic Modulus Using Load and Displacement Sensing Indentation Experiments,” Journal of Materials Research 7, no. 6 (1992): 1564–1583, 10.1557/jmr.1992.1564.

[35] P. Venezuela , M. Lazzeri , and F. Mauri , “Theory of Double‐Resonant Raman Spectra in Graphene: Intensity and Line Shape of Defect‐Induced and Two‐Phonon Bands,” Physical Review B 84, no. 3 (2011): 035433, 10.1103/physrevb.84.035433.

[36] A. Eckmann , A. Felten , A. Mishchenko , et al., “Probing the Nature of Defects in Graphene by Raman Spectroscopy,” Nano Letters 12, no. 8 (2012): 3925–3930, 10.1021/nl300901a. 22764888

[37] C. Neumann , S. Reichardt , P. Venezuela , et al., “Raman Spectroscopy as Probe of Nanometre‐Scale Strain Variations in Graphene,” Nature Communications 6, no. 1 (2015): 8429, 10.1038/ncomms9429. PMC4598719 26416349

[38] J. E. Lee , G. Ahn , J. Shim , Y. S. Lee , and S. Ryu , “Optical Separation of Mechanical Strain From Charge Doping in Graphene,” Nature Communications 3, no. 1 (2012): 1024, 10.1038/ncomms2022. 22929781

[39] T. M. G. Mohiuddin , A. Lombardo , R. R. Nair , et al., “Uniaxial Strain in Graphene by Raman Spectroscopy: G Peak Splitting, Grüneisen Parameters, and Sample Orientation,” Physical Review B 79, no. 20 (2009): 205433, 10.1103/physrevb.79.205433.

[40] G. Ahn and S. Ryu , “Reversible Sulfuric Acid Doping of Graphene Probed by in‐Situ Multi‐Wavelength Raman Spectroscopy,” Carbon 138 (2018): 257–263, 10.1016/j.carbon.2018.05.065.

[41] R. Larciprete , S. Ulstrup , P. Lacovig , et al., “Oxygen Switching of the Epitaxial Graphene–Metal Interaction,” ACS Nano 6, no. 11 (2012): 9551–9558, 10.1021/nn302729j. 23051045

[42] R. Blume , P. R. Kidambi , B. C. Bayer , et al., “The Influence of Intercalated Oxygen on the Properties of Graphene on Polycrystalline Cu Under Various Environmental Conditions,” Physical Chemistry Chemical Physics 16, no. 47 (2014): 25989–26003, 10.1039/c4cp04025b. 25356600

[43] C. Gong , G. Lee , B. Shan , E. M. Vogel , R. M. Wallace , and K. Cho , “First‐Principles Study of Metal–Graphene Interfaces,” Journal of Applied Physics 108, no. 12 (2010): 123711, 10.1063/1.3524232.

[44] M. Nonnenmacher , M. P. O'Boyle , and H. K. Wickramasinghe , “Kelvin Probe Force Microscopy,” Applied Physics Letters 58, no. 25 (1991): 2921–2923, 10.1063/1.105227.

[45] P. A. Fernández Garrillo , B. Grévin , N. Chevalier , and Ł. Borowik , “Calibrated Work Function Mapping by Kelvin Probe Force Microscopy,” Review of Scientific Instruments 89, no. 4 (2018): 043702, 10.1063/1.5007619. 29716375

[46] D. Ziegler , P. Gava , J. Güttinger , et al., “Variations in the Work Function of Doped Single‐ and Few‐Layer Graphene Assessed by Kelvin Probe Force Microscopy and Density Functional Theory,” Physical Review B 83, no. 23 (2011): 235434, 10.1103/physrevb.83.235434.

[47] F. Long , P. Yasaei , R. Sanoj , et al., “Characteristic Work Function Variations of Graphene Line Defects,” ACS Applied Materials & Interfaces 8, no. 28 (2016): 18360–18366, 10.1021/acsami.6b04853. 27356179

[48] E. Khan , T. S. Rahman , and S. Subrina , “Electronic Structure of Bilayer Graphene Physisorbed on Metal Substrates,” Journal of Applied Physics 120, no. 18 (2016): 185101, 10.1063/1.4966612.

[49] Y. Sui and J. Appenzeller , “Screening and Interlayer Coupling in Multilayer Graphene Field‐Effect Transistors,” Nano Letters 9, no. 8 (2009): 2973–2977, 10.1021/nl901396g. 19639984

[50] R. G. Forbes , “Extraction of Emission Parameters for Large‐Area Field Emitters, Using a Technically Complete Fowler–Nordheim‐Type Equation,” Nanotechnology 23, no. 9 (2012): 095706, 10.1088/0957-4484/23/9/095706. 22327471

[51] F. Liu , N. A. Moody , K. L. Jensen , et al., “Single Layer Graphene Protective Gas Barrier for Copper Photocathodes,” Applied Physics Letters 110, no. 4 (2017): 041607, 10.1063/1.4974738.

[52] K. Hata , A. Takakura , and Y. Saito , “Field Emission Microscopy of Adsorption and Desorption of Residual Gas Molecules on a Carbon Nanotube Tip,” Surface Science 490, no. 3 (2001): 296–300, 10.1016/S0039-6028(01)01338-3.

[53] G. Chen , W. Wang , J. Peng , et al., “Screening Effects on Field Emission From Arrays of (5,5) Carbon Nanotubes: Quantum Mechanical Simulations,” Physical Review B 76, no. 19 (2007): 195412, 10.1103/physrevb.76.195412.

[54] R. C. Smith and S. R. P. Silva , “Maximizing the Electron Field Emission Performance of Carbon Nanotube Arrays,” Applied Physics Letters 94, no. 13 (2009): 133104, 10.1063/1.3097239.

[55] G. Kresse and J. Furthmüller , “Efficient Iterative Schemes for Ab Initio Total‐Energy Calculations Using a Plane‐Wave Basis Set,” Physical Review B 54, no. 16 (1996): 11169–11186, 10.1103/physrevb.54.11169. 9984901

[56] J. P. Perdew , K. Burke , and M. Ernzerhof , “Generalized Gradient Approximation Made Simple,” Physical Review Letters 77, no. 18 (1996): 3865–3868, 10.1103/physrevlett.77.3865. 10062328

[57] L. Bengtsson , “Dipole Correction for Surface Supercell Calculations,” Physical Review B 59, no. 19 (1999): 12301–12304, 10.1103/physrevb.59.12301.

