# Supplementary material for: Field Emission Enhancement in Graphene/Cu Cathodes Through Laser‐Shock‐Induced Modulation of Geometry and Work Function
Source: Adv Sci (Weinh). 2026 Nov 10:e78333. Online ahead of print. doi: 10.1002/advs.78333 (PMC13750013; doi:10.1002/advs.78333)
Supplement: Supplementary file 1 [file ADVS-9999-e78333-s001.pdf]

Supporting Information

Field Emission Enhancement in Graphene/Cu Cathodes through Laser-Shock-Induced Modulation of Geometry and Work Function

Ximei Xu[a], Haonan Tao[a], Wenming Li[a], Xuyin Li[a], Jiacong Lin[a], Mingyang Yang[a,c] and Ming Zhou[a,b,c]**

a. State Key Laboratory of Clean and Efficient Turbomachinery Power Equipment, Department of Mechanical Engineering, Tsinghua University, Beijing 100084, China

b. Key Laboratory for Advanced Materials Processing Technology, Ministry of Education, Beijing 100084, China

c. Beijing Key Laboratory of Atomic-Scale Precision Manufacturing and Cross-Scale Device Fabrication, Department of Mechanical Engineering, Tsinghua University, Beijing 100084, China

Corresponding authors:
Ming Zhou, Email: zhouming@tsinghua.edu.cn
Mingyang Yang, Email: yangmy@mail.tsinghua.edu.cn

Contents

Notes

Note S1. Estimation of laser shock pressure and characteristic strain rate

Note S2. Raman vector decomposition of the centroid shift

Note S3. Quantification of the effective treated area fraction from InLens SEM images

Note S4. Thermal drift check during nanoindentation

Figures

Figure S1. STEM–EDS line scan across the graphene/Cu interface in the protrusion top region

Figure S2. 60 kV TEM images of graphene after laser shock microforming

Figure S3. Bundled planar defects in deformed Cu near the protrusion sidewall region

Figure S4. Surface relief effects and modulus output in nanoindentation of graphene/Cu foils

Figure S5. Regional SEM–EDS comparison of relative C, O and Cu signals in graphene/Cu after laser shock

Figure S6. Enlarged structural views of the DFT models M1–M6

Figure S7. Field emission from independently fabricated bilayer graphene/Cu cathodes

Figure S8. Field emission and Fowler–Nordheim analysis of a separately optimized monolayer graphene/Cu cathode

Figure S9. Geometry model and mesh refinement for electrostatic simulation

Figure S10. Field emission measurements of bare Cu control cathodes

Figure S11. Field emission from closely packed nanostructured cathodes

Figure S12. Workflow for extracting the effective treated area fraction from InLens SEM images

Figure S13. Ag reference spectra and secondary electron cutoff extraction in UPS

Figure S14. Ultraviolet photoelectron spectroscopy spectra and assessment of the secondary electron cutoff

Figure S15. KPFM reference measurement on an Au surface

Figure S16. KPFM mapping and SEM morphology of pit structures formed by laser shock in graphene/Cu

Tables

Table S1. Grouped peak fitting results for the high-resolution C 1s and O 1s XPS spectra

Table S2. Simplified DFT models for comparing representative factors affecting the work function

Notes

Note S1. Estimation of laser shock pressure and characteristic strain rate

Laser shock loading was generated by confined laser-induced plasma expansion. The peak pressure was estimated using the confined plasma model developed for laser-induced plasmas in confined geometry[1]. In this model, the pressure estimate depends on the incident laser power density and the reduced shock impedance of the media that transmit the shock toward the graphene/Cu foil[2]. Local pressure and strain rate can vary across the template owing to nonuniform contact, sidewall friction and local plastic flow of the Cu foil. We therefore used one representative parameter set to estimate the pressure and strain rate scale of the process.

$$p_{\mathrm{peak}} = 0.01\left(\frac{\alpha}{2\alpha + 3}\right)^{1/2} Z^{1/2} {I_0}^{1/2}$$

where p_{peak} is the peak pressure in GPa, I_0 is the laser power density in GW cm^{-2}, Z is the reduced shock impedance in g cm^{-2} s^{-1}, and α is the fraction of internal energy converted into thermal energy of the plasma. For this estimate, α was taken as 0.1. The reduced shock impedance is defined as

$$\frac{2}{Z} = \frac{1}{Z_{\mathrm{Al}}} + \frac{1}{Z_{\mathrm{Cu}}}$$

For the representative processing condition, we estimated the reduced impedance using the Al absorption layer and the Cu foil. Representative longitudinal acoustic impedance values for rolled Al and rolled Cu[3] are $Z_{\mathrm{Al}} = 1.73 \times 10^6$ and $Z_{\mathrm{Cu}} = 4.47 \times 10^6$ g cm^{-2} s^{-1}. The pulse energy was $E_{\mathrm{p}} = 260$ mJ, the pulse duration was $\tau = 9$ ns, and the laser spot diameter was $D = 0.88$ mm. The laser power density was calculated as

$$I_0 = \frac{4E_{\mathrm{p}}}{\pi\tau D^2}$$

Substitution of these values gives

$$p_{\mathrm{peak}} \approx 6.1\ \ \mathrm{GPa}$$

The characteristic strain rate was estimated from a characteristic deformation scale inferred from the protrusion geometry and an effective loading time. The protrusions formed with the sapphire template have a height of approximately 400 nm and a lateral feature size of approximately 0.6–1.0 μm, corresponding to a dimensionless geometric deformation scale of approximately 0.4–0.7. The effective loading time was taken as 20–100 ns, comparable to the laser pulse duration and the extended pressure duration expected under transparent confinement. This gives a characteristic strain-rate scale of approximately 10^6–10^7 s^{-1}. Overall, the estimated pressure and strain-rate scales are

consistent with gigapascal-level shock loading and plastic deformation of Cu at high strain rates during laser shock processing[4].

Note S2. Raman vector decomposition of the centroid shift

Raman spectra were not collected from identical physical locations before and after laser shock. Therefore, the pristine measurements and laser-shocked measurements were treated as two statistical data sets.

The centroids of the pooled pristine data set (1-p and 2-p) and the pooled laser-shocked data set (1-L and 2-L) were located at (ω_G, ω_{2D}) = (1583.41, 2659.69) and (1588.64, 2647.01) cm^{-1}, respectively. The corresponding centroid shift was ($\Delta\omega_G$, $\Delta\omega_{2D}$) = (+5.23, −12.68) cm^{-1}. Following the Raman correlation method[5,6], the shift vector was decomposed as $\boldsymbol{v} = a(1, 2.45) + b(1, 0.70)$, where a and b are scalar coefficients along the reference directions associated with strain and doping, respectively. This decomposition gives $a = -9.34$ and $b = +14.57$. The negative a and positive b values correspond to a tensile strain component and a tendency toward hole doping, respectively, and this interpretation remains unchanged for slopes of the strain axis within the commonly used range of 2.2–2.5.

To test whether this conclusion depends on arbitrary point matching, we further decomposed all possible vectors from the pristine data set to the laser-shocked data set. For the pooled data, all 15 × 15 = 225 vectors gave $a < 0$ and $b > 0$. The same result was obtained when the monolayer and bilayer subsets were analyzed separately: all vectors from 1-p to 1-L and all vectors from 2-p to 2-L gave the same sign combination. Thus, the inferred tensile strain component and hole doping tendency do not depend on a particular point pairing scheme.

Note S3. Quantification of the effective treated area fraction from InLens SEM images

The effective treated area fraction, f_{eff}, was quantified from raw InLens SEM images to compare the relative coverage of regions modified by laser shock among cathodes processed under different conditions. InLens imaging was selected because it emphasizes near-surface secondary electron contrast and is therefore sensitive to local relief, protrusions and edge features under matched imaging conditions[7–9]. In our SEM observations, these micro- and nanoscale features showed stronger contrast against the relatively flat background in InLens images than in images acquired with the conventional secondary electron detector. At the relatively large field of view used here, many of these features appeared collectively as a broad bright domain. This bright domain was defined as the effective treated area in the InLens SEM image analysis.

Each SEM image was converted to grayscale, and the grayscale intensity was linearly normalized to the range of 0–1. The bright domain was segmented by thresholding the normalized grayscale image[10]. The final threshold was set to $T = 0.525$. This value was

selected from threshold sensitivity tests and was further validated by detailed comparison of the segmented bright domains with higher magnification InLens SEM images of the corresponding regions. Pixels with grayscale intensity above T were assigned to the segmented bright domain.

The effective treated area fraction, f_{eff}, was calculated as the ratio of the segmented bright domain area to the total SEM image area. This fraction provides a consistent relative metric for comparing the macroscopic coverage of regions modified by laser shock among different cathodes. In the field emission analysis, a larger f_{eff} indicates a larger relative surface fraction containing features that can serve as candidate emission sites, such as local relief, edges and sharp geometric features.

Note S4. Thermal drift check during nanoindentation

Nanoindentation was performed under load control as described in Methods. To verify that the representative load–displacement curves were not dominated by thermal drift, we independently evaluated the displacement as a function of time within the low load drift hold segment. This segment was recorded after unloading to approximately 0.1 P_{max} and before final unloading. During this segment, the load was close to constant and was therefore suitable for assessing displacement drift.

For representative 1-p and 1-L indentations, the endpoint drift rates were $|v_{end}| \approx 0.0063$ nm s^{-1} and 0.0025 nm s^{-1}, respectively. Conservative upper bounds estimated from the difference between the maximum and minimum displacement during the same segment were approximately 0.0108 nm s^{-1} and 0.0106 nm s^{-1}, respectively. These values are far below the 0.05 nm s^{-1} level often used as a practical benchmark for low thermal drift in nanoindentation measurements[11].

For maximum indentation depths of approximately 0.5–0.8 μm, the measured drift corresponds to a displacement change much smaller than h_{max}. Over the approximately 75 s drift hold segment, the endpoint drift rates correspond to less than approximately 0.5 nm of displacement change, while the conservative estimates based on the full displacement range correspond to less than approximately 0.9 nm. The same analysis was applied to the nanoindentation data points included in the hardness comparison, and all inspected drift hold segments remained below the practical benchmark.

Figures

Figure S1. STEM–EDS line scan across the graphene/Cu interface in the protrusion top region

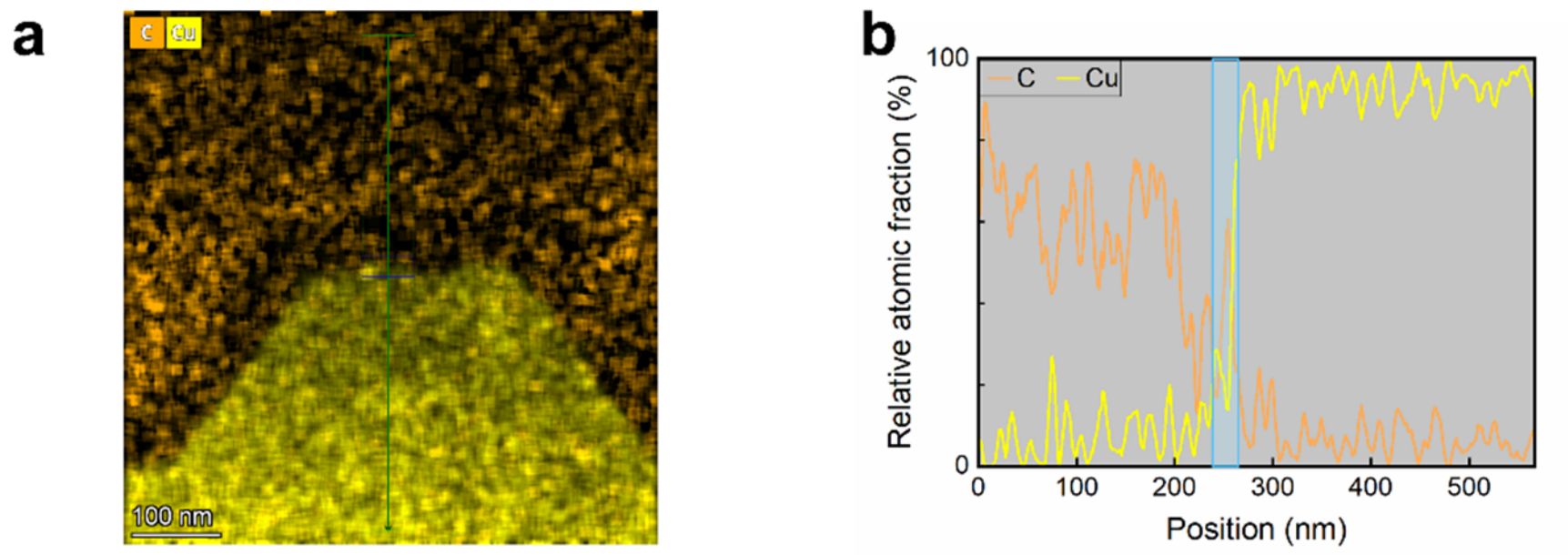

(a) Cross-sectional STEM–EDS elemental overlay showing the distributions of C and Cu near the protrusion top region of laser-shocked graphene/Cu. The vertical line marks the scan path used for the elemental profile in (b).

(b) Relative C and Cu EDS atomic fraction profiles extracted along the line marked in (a). The blue boxed region highlights the position near the interface where the Cu signal decreases locally while the C signal shows a local increase. This local increase is consistent with carbon enrichment near the Cu interface and supports a local carbon signal associated with graphene.

Figure S2. 60 kV TEM images of graphene after laser shock microforming.

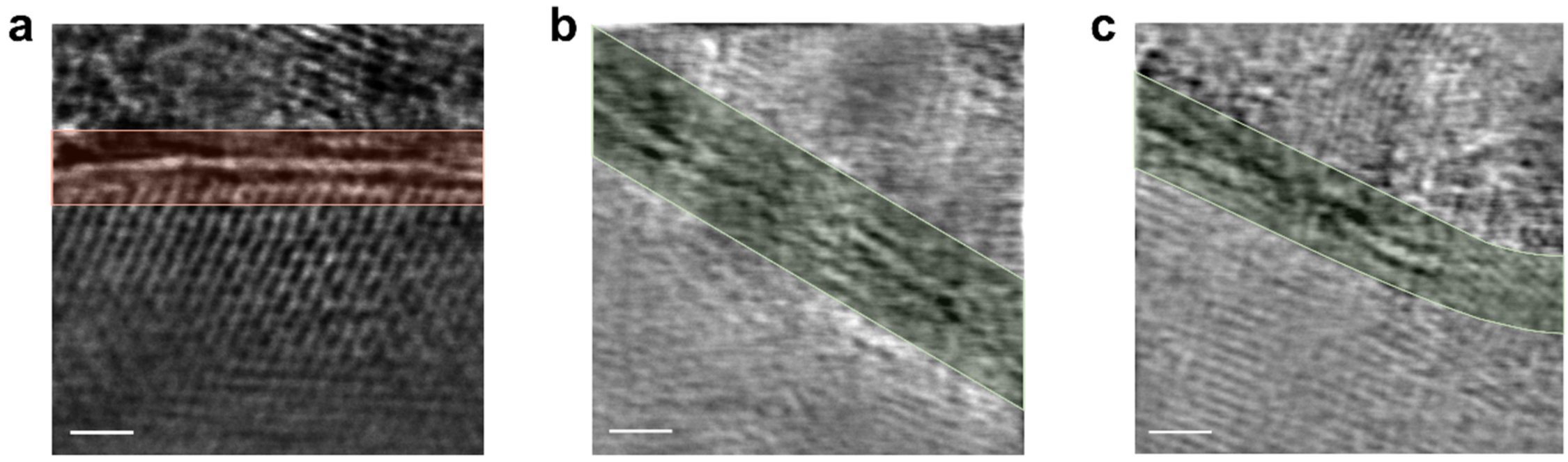

(a) TEM image acquired at 60 kV from the flat graphene/Cu substrate region, where graphene remains relatively continuous along the Cu surface.

(b, c) TEM images acquired at 60 kV from the protrusion sidewall and root regions, respectively. In these deformed regions, the continuity of graphene is strongly reduced, with local fragmentation. The 60 kV images show the same spatial trend as the 300 kV TEM observations while reducing damage to graphene caused by the electron beam during imaging. Colored guide lines mark the graphene interface region. Scale bars, 1 nm.

Figure S3. Bundled planar defects in deformed Cu near the protrusion sidewall region.

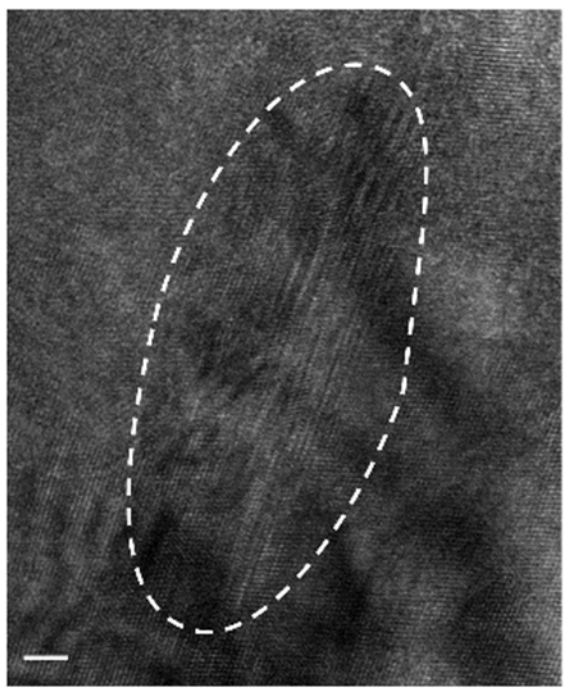

High-resolution TEM image from the protrusion sidewall region of graphene/Cu after laser shock microforming. The dashed outline marks bundled, closely spaced planar defects in the Cu substrate, consistent with defects related to stacking faults generated during plastic deformation of Cu at high strain rates. Scale bar, 2 nm.

Figure S4. Surface relief effects and modulus output in nanoindentation of graphene/Cu foils.

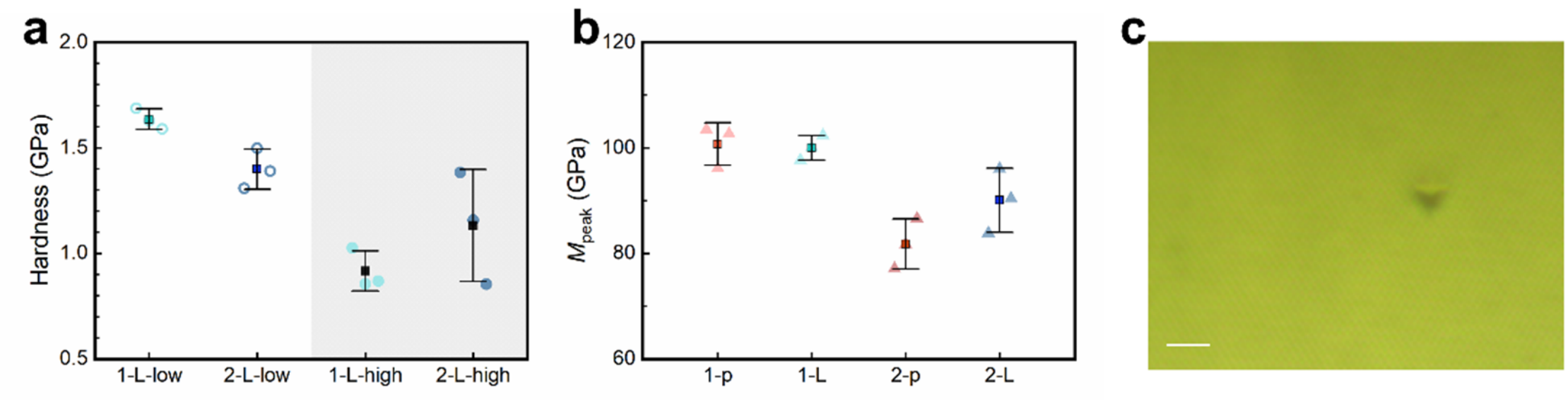

(a) Apparent hardness measured in regions with low and high surface relief in 1-L and 2-L samples. Regions with low relief show smaller scatter and were used for the main hardness comparison in Figure 2i. Regions with high relief show larger dispersion, consistent with the known sensitivity of nanoindentation measurements to local surface morphology, roughness and contact geometry[12].

(b) Statistical comparison of the modulus output at maximum load, M_{peak}, for 1-p, 1-L, 2-p and 2-L samples. M_{peak} denotes the indentation modulus value at maximum load. No systematic change in M_{peak} was observed between pristine and laser-shocked samples.

(c) Optical image of a residual indent on a graphene/Cu foil after laser shock. The residual indent spans several surface relief features; the indentation measurement is therefore treated as an averaged response near the surface within the microformed region. Squares and error bars denote means ± s.d. (n = 3 independent indents). Scale bar, 5 μm.

Figure S5. Regional SEM–EDS comparison of relative C, O and Cu signals in graphene/Cu after laser shock.

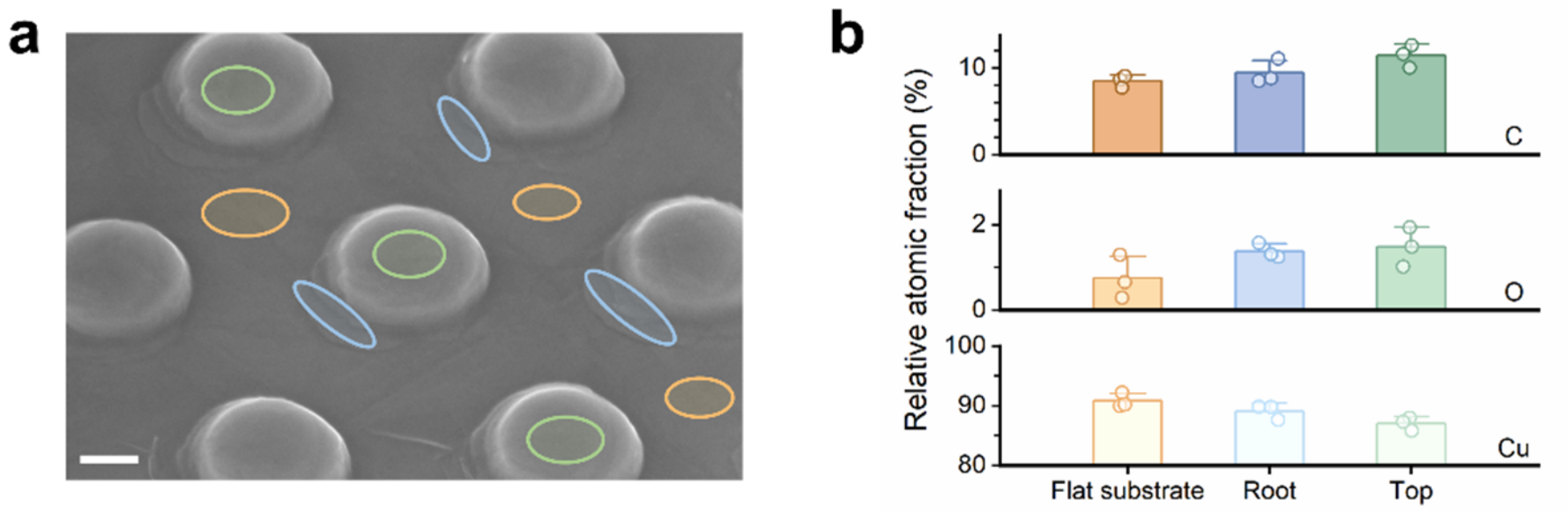

(a) SEM image showing EDS analysis regions selected from the flat substrate region (orange), protrusion root region (blue) and protrusion top region (green). Scale bar, 200 nm.

(b) Relative atomic fractions of C, O and Cu extracted from the selected regions (mean ± s.d., n = 3). Compared with the flat substrate region, the protrusion root and top regions show higher relative O signals, accompanied by a locally enhanced C signal at the protrusion top and a corresponding decrease in the relative Cu fraction. This regional trend supports relative oxygen enrichment in graphene/Cu after laser shock and is consistent with the XPS results discussed in the main text.

Figure S6. Enlarged structural views of the DFT models M1–M6.

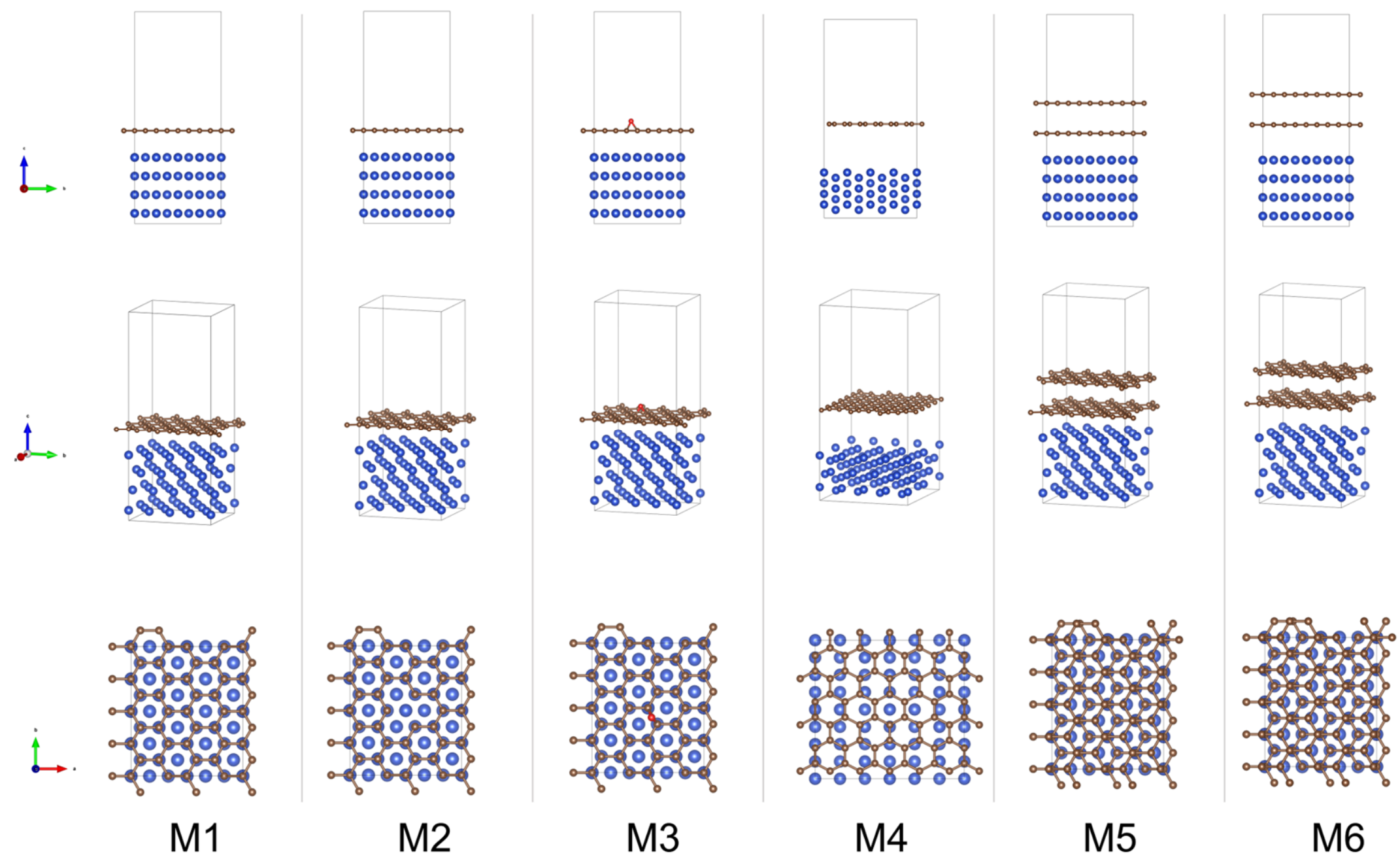

Columns correspond to M1–M6, and the top, middle and bottom rows show side, perspective and top views, respectively. M1 and M5 are the monolayer and bilayer graphene/Cu(111) reference configurations. Relative to M1, M2 contains a graphene vacancy, M3 includes a C–O bridge configuration on graphene, and M4 uses a stepped Cu(211) surface. Relative to M5, M6 has an increased graphene/Cu separation while retaining the graphene interlayer separation. Brown, blue and red spheres represent C, Cu and O atoms, respectively.

Figure S7. Field emission from independently fabricated bilayer graphene/Cu cathodes.

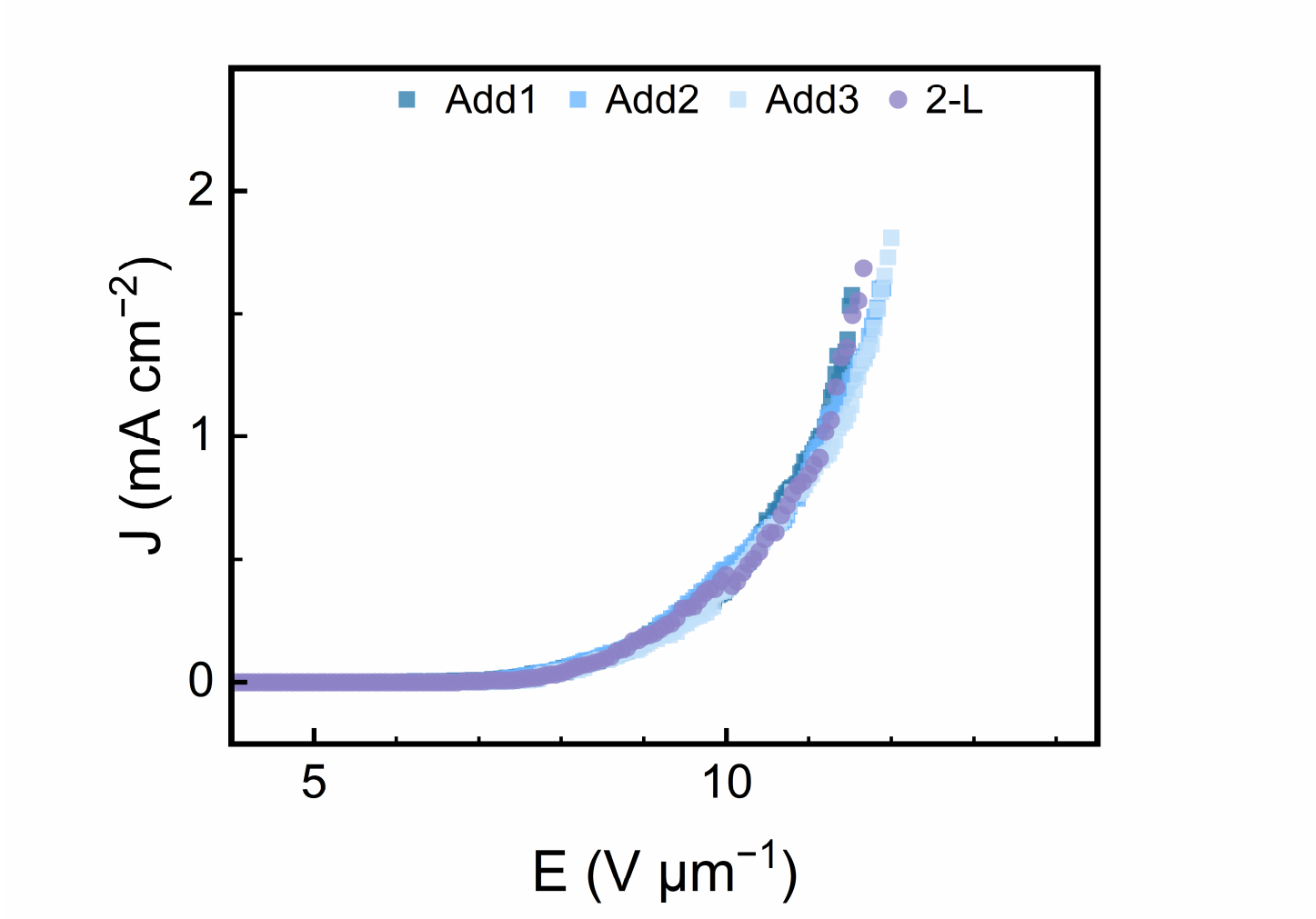

J–*E* curves of the original 2-L cathode and three additional cathodes (Add1–Add3) prepared under the same processing conditions. The turn-on field, E_{on}, defined at $J = 10$ μA cm^{-2}, is 7.35 ± 0.16 V μm^{-1} (n = 4).

Figure S8. Field emission and Fowler–Nordheim analysis of a separately optimized monolayer graphene/Cu cathode.

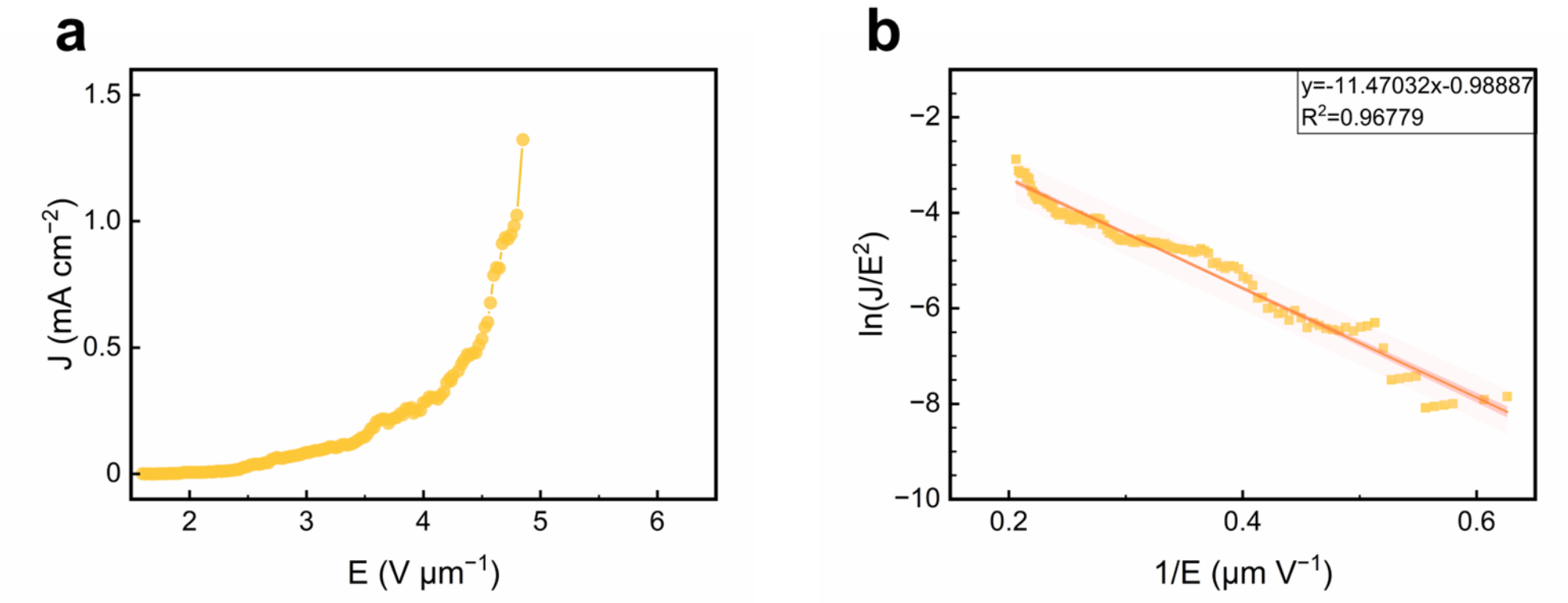

(a) Current density–electric field (J–E) curve.

(b) Corresponding Fowler–Nordheim (FN) plot, shown as $\ln(J/E^2)$ versus $1/E$, using numerical values of J in mA cm^{-2} and E in V μm^{-1}. The solid line shows the linear fit over the full recorded positive-current range, $J = 0.001$–1.32267 mA cm^{-2} ($E = 1.59750$–4.84833 V μm^{-1}). The fit gives $S_{FN} = -11.47032$ V μm^{-1}, $C = -0.98887$, $R^2 = 0.96779$, and a residual sum of squares of 6.33348. Using the fixed reference work function $\Phi_{ref} = 4.25$ eV and $\beta_{app} = -b\Phi_{ref}^{3/2}/S_{FN}$, with $b = 6.830890\times10^3$ $eV^{-3/2}$ V μm^{-1}, gives $\beta_{app} \approx 5\times10^3$.

Figure S9. Geometry model and mesh refinement for electrostatic simulation.

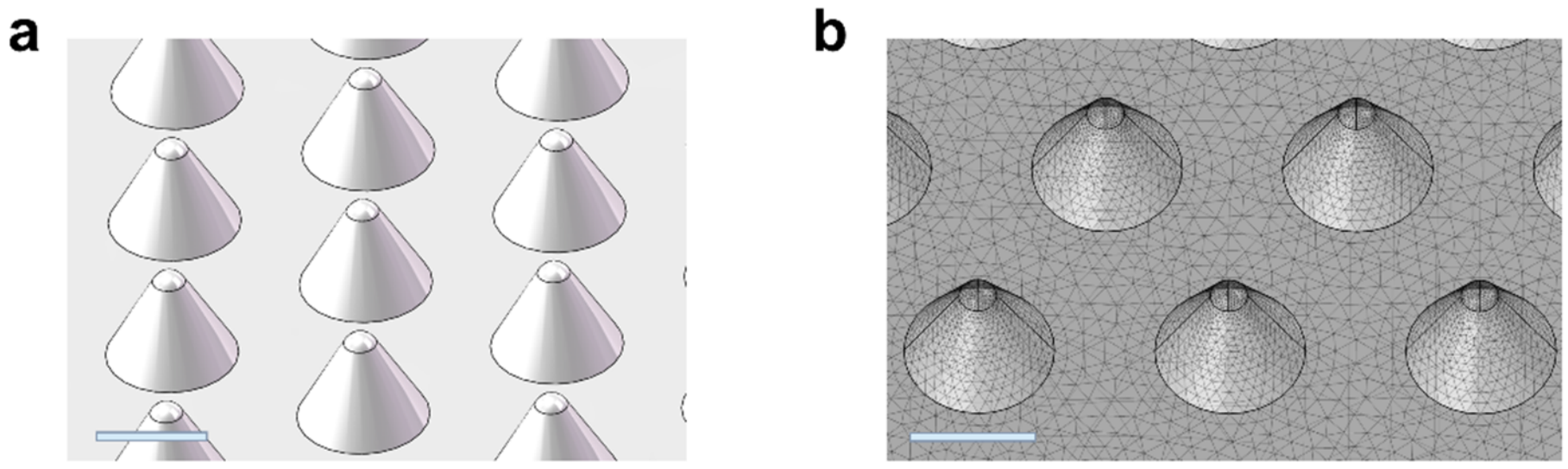

(a) Representative cathode geometry used for electrostatic simulation, constructed from SEM and AFM observations of the graphene/Cu protrusion array formed by laser shock. A planar anode was placed 300 µm above the cathode, and a 5 kV bias was applied between the electrodes, consistent with the field emission measurement conditions.

(b) Finite element mesh of the protrusion array, showing local refinement near the protrusion apex and top rim where the electric field is most concentrated. Scale bars, 500 nm.

Figure S10. Field emission measurements of bare Cu control cathodes.

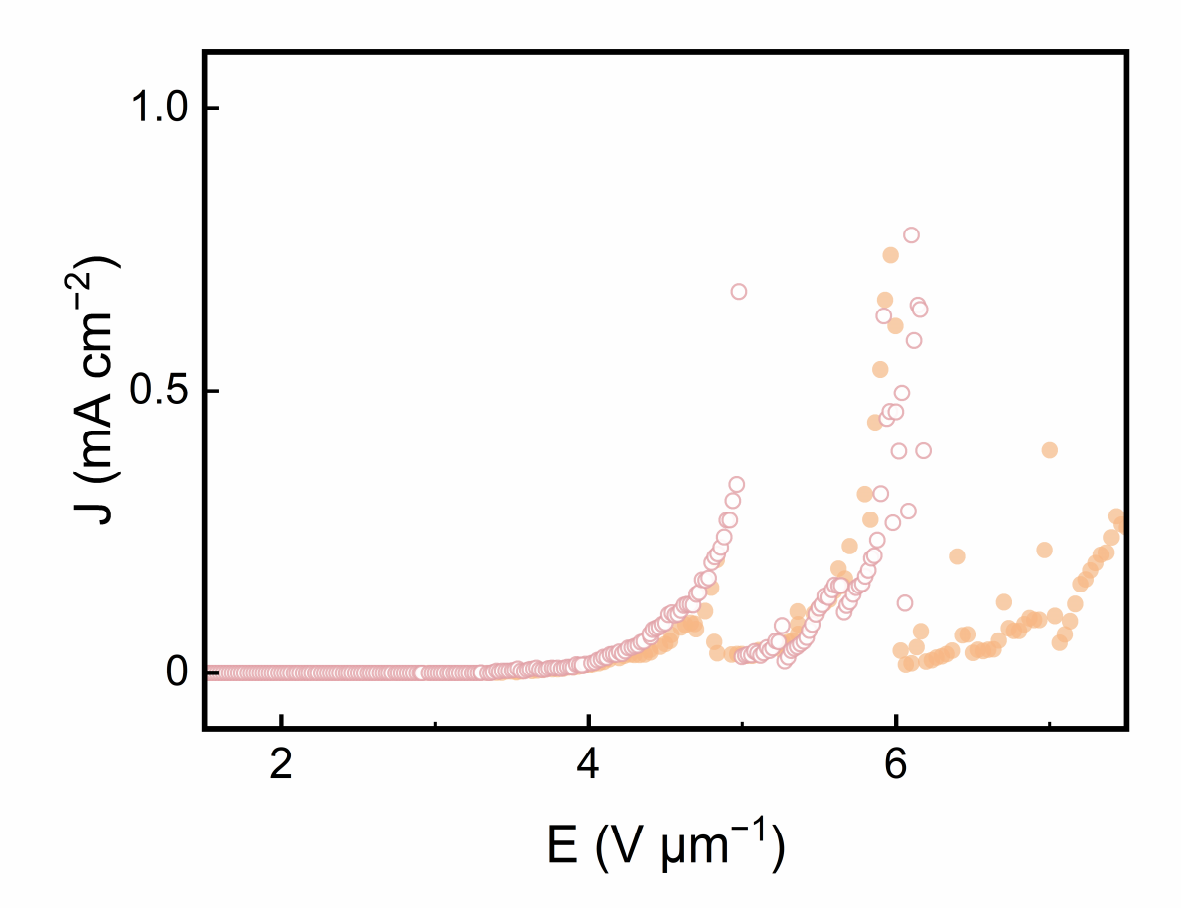

Current density–electric field (*J*–*E*) curves of two laser-shocked bare Cu cathodes processed using the same templates and laser conditions as graphene/Cu. Pristine Cu showed no detectable emission below 15 V μm^{-1}. At the higher laser power density, bare Cu produced measurable emission but exhibited large current variations during voltage sweeps.

Figure S11. Field emission from closely packed nanostructured cathodes.

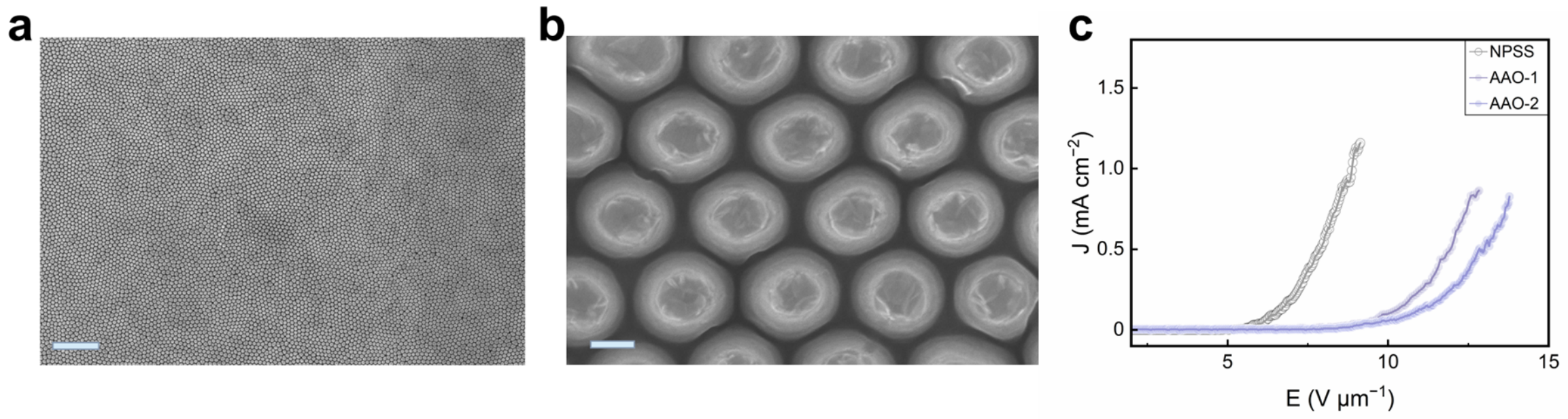

(a) Low magnification SEM image of a closely packed nanostructured graphene/Cu cathode fabricated using an anodic aluminum oxide (AAO) template.

(b) Higher magnification SEM image showing the closely packed nanostructure.

(c) Comparison of field emission J–E characteristics between a graphene/Cu cathode patterned with a periodic discrete protrusion array fabricated using an NPSS template and closely packed nanostructured graphene/Cu cathodes fabricated using AAO templates. Under the same laser processing and measurement conditions, the cathode with discrete protrusions shows a lower turn-on field than the closely packed nanostructured cathodes. The dense nanostructure increases the number of potential emission sites but reduces the spacing between neighboring emitters, which can strengthen electrostatic screening. Practical optimization therefore requires a balance between hot spot density and screening. Scale bars, 5 μm in a and 200 nm in b.

Figure S12. Workflow for extracting the effective treated area fraction from InLens SEM images.

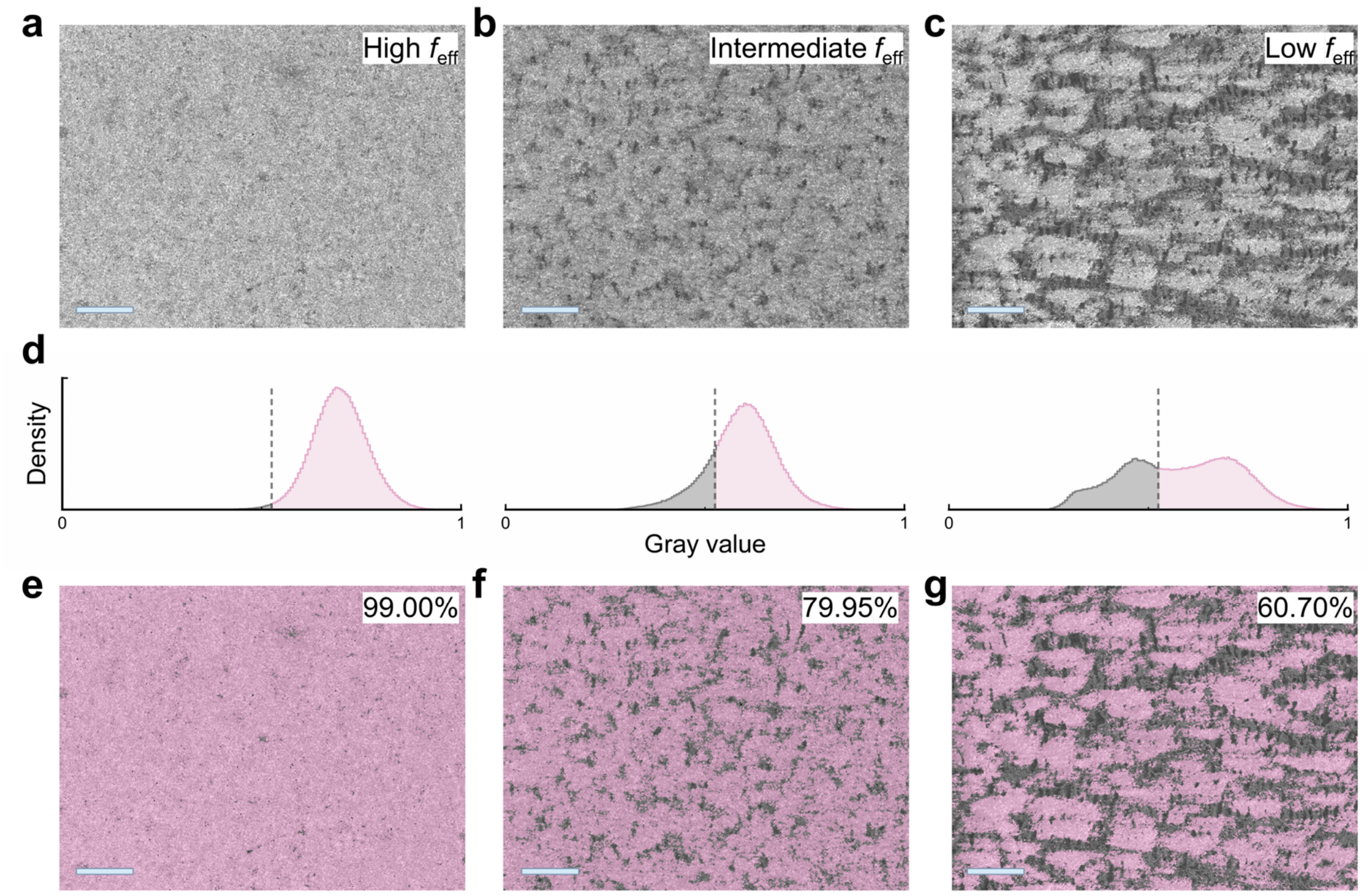

(a–c) Raw InLens SEM images of cathodes with high, intermediate and low effective treated area fractions, f_{eff}.

(d) Normalized grayscale intensity histograms of the corresponding images. The dashed vertical line marks the common threshold, $T = 0.525$, on the 0–1 grayscale intensity scale. The threshold was selected from sensitivity tests and validated by comparing the segmented bright domains with higher magnification InLens SEM images of the corresponding regions.

(e–g) Segmentation overlays of the same fields of view as (a–c), generated using the same threshold. Pixels with grayscale intensity above T were assigned to the segmented bright domains, shown in pink, which were used to define the effective treated area and attributed to regions modified by laser shock. f_{eff} is used here as a relative InLens SEM contrast metric for comparing the areal coverage of regions modified by laser shock across cathodes. Scale bars, 50 µm.

Figure S13. Ag reference spectra and secondary electron cutoff extraction in UPS.

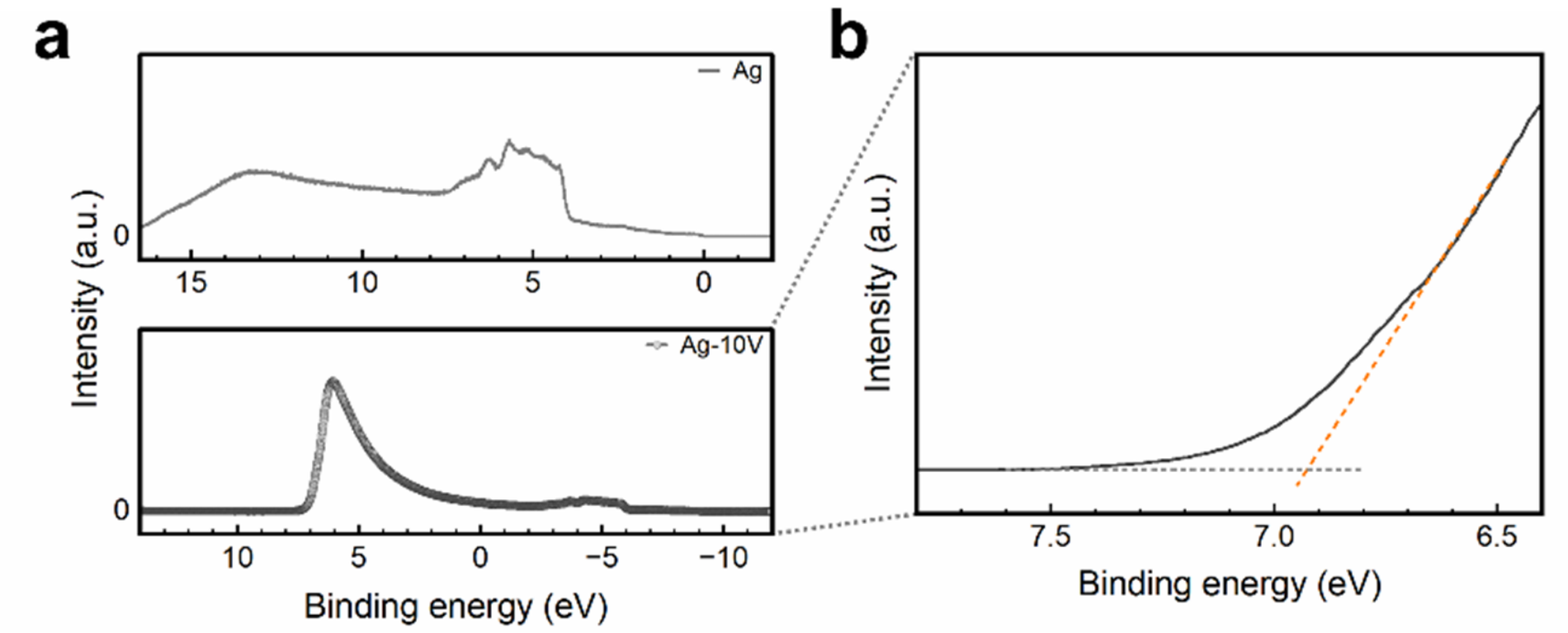

(a) UPS spectra of the Ag reference acquired without bias and under the same 10 V sample bias using a He ultraviolet light source (*hν* = 21.22 eV). The unbiased spectrum was used to monitor the Fermi edge position, whereas the biased spectrum was used for secondary electron cutoff (SECO) extraction.

(b) Enlarged SECO region of the biased Ag reference spectrum, showing the linear extrapolation used to determine E_{SECO}. Their intersection defines the cutoff position, E_{SECO}.

Figure S14. Ultraviolet photoelectron spectroscopy spectra and assessment of the secondary electron cutoff.

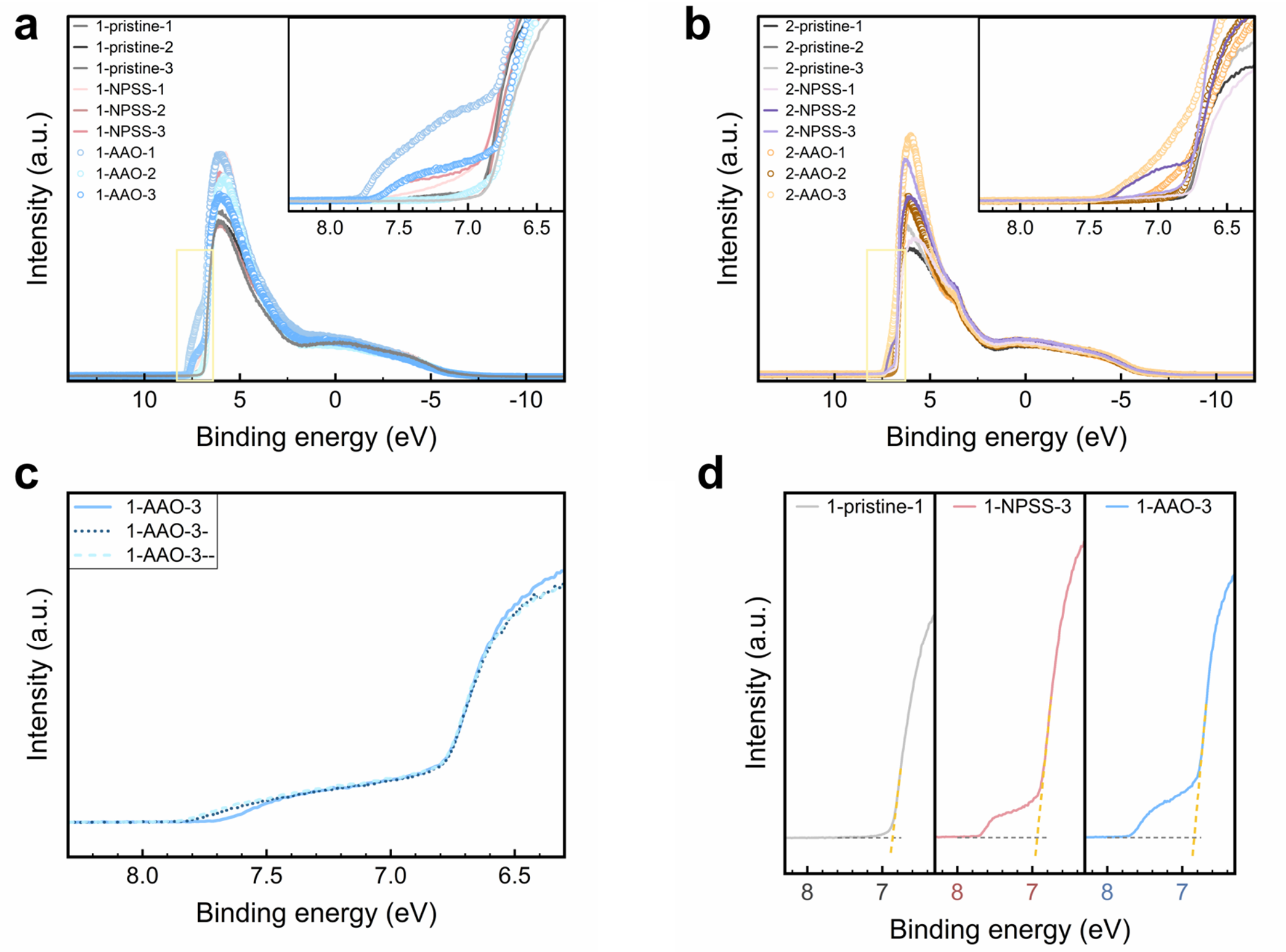

(a–b) Complete UPS spectra of (a) monolayer and (b) bilayer graphene/Cu, including pristine specimens and specimens laser-shocked using AAO or NPSS templates. Insets show enlarged secondary electron cutoff (SECO) regions. Three distinct specimens were measured for each condition and graphene layer number, with each treated specimen separately laser-shocked.

(c) SECO regions from three acquisitions of the same representative specimen, 1-AAO-3, showing the shoulder feature in all three spectra.

(d) Comparison of the SECO regions of a pristine specimen without a pronounced shoulder (1-pristine-1) and laser-shocked specimens with pronounced shoulders (1-NPSS-3 and 1-AAO-3), shown over the same binding-energy range. Linear extrapolations of the selected rising segments and the corresponding baselines illustrate determination of the apparent cutoff from their intersections. For spectra containing shoulders, extrapolation of only the later, dominant rise bypasses the preceding onset and does not capture the complete onset profile, limiting work function comparisons based on a single extrapolated cutoff.

Figure S15. KPFM reference measurement on an Au surface.

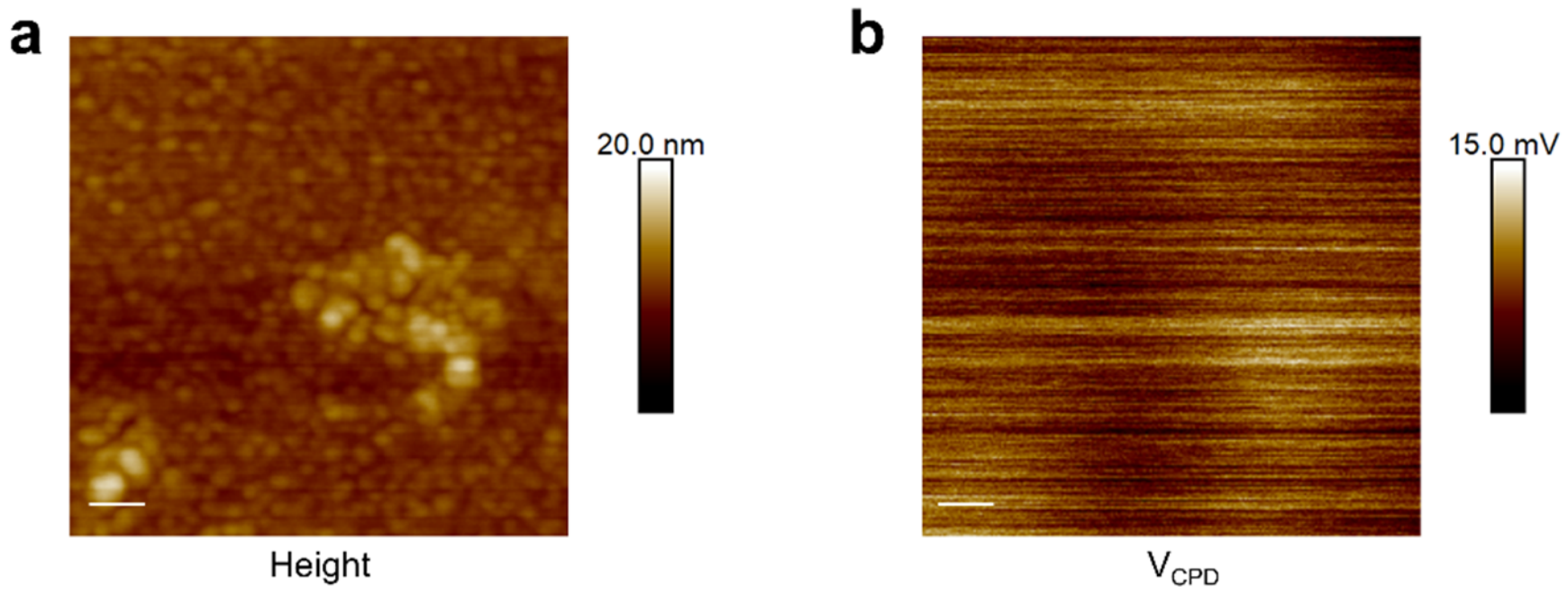

(a) Topography of a freshly prepared Au reference surface measured during the KPFM measurement sequence.

(b) Corresponding CPD map acquired with the same probe during the graphene/Cu measurement sequence. The Au reference was used as a check of the probe condition and scan stability during the measurement sequence[13]. Scale bars, 100 nm.

Figure S16. KPFM mapping and SEM morphology of pit structures formed by laser shock in graphene/Cu.

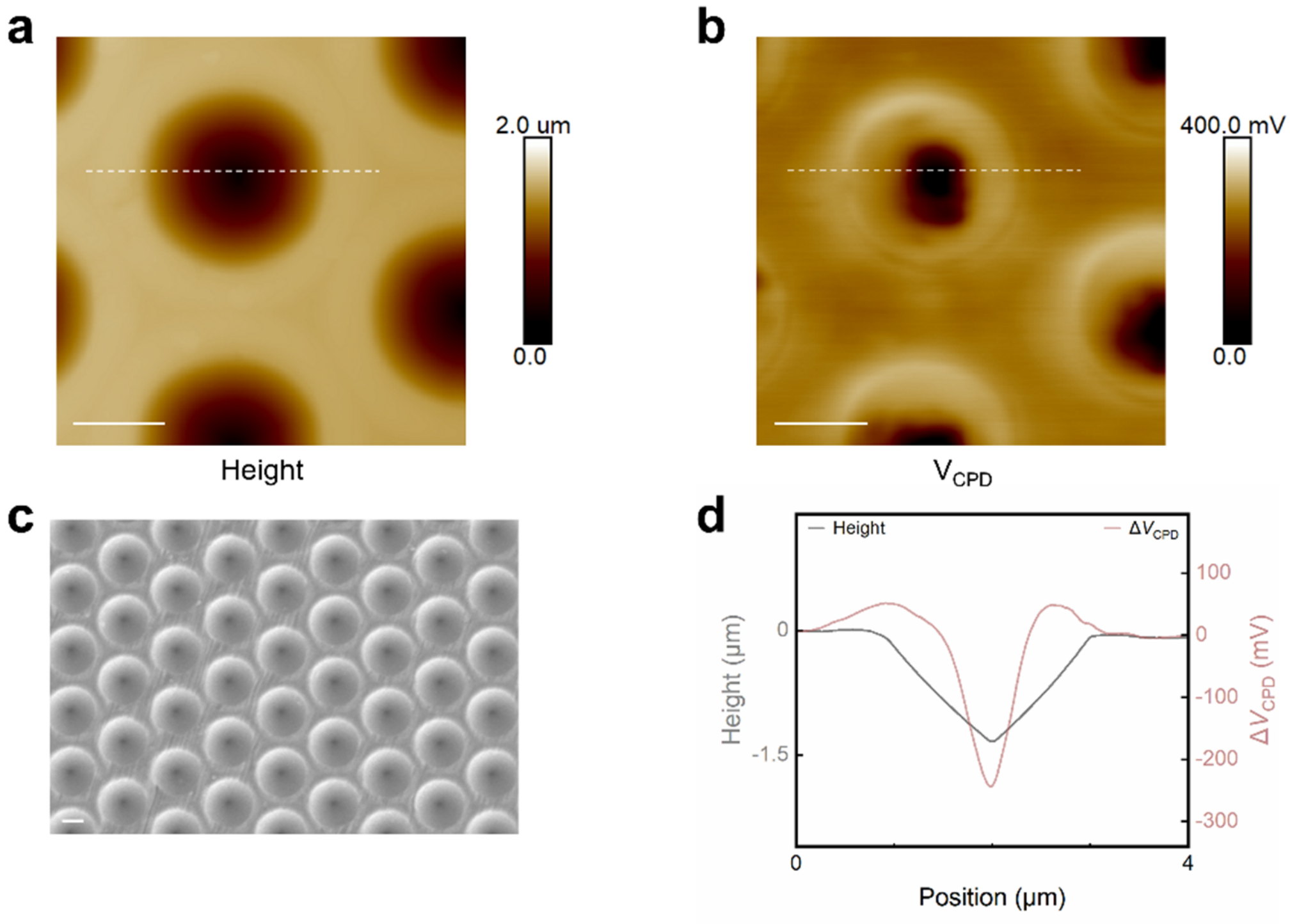

(a) AFM topography map of a pit morphology formed by laser shock in graphene/Cu.

(b) Corresponding V_{CPD} map of the same region.

(c) SEM overview of the pit array morphology formed by laser shock in graphene/Cu.

(d) Height and ΔV_{CPD} profiles extracted along the dashed lines in (a) and (b). The pit region shows clear local V_{CPD} contrast relative to the surrounding flatter substrate region, and this contrast is spatially associated with the concave surface morphology, indicating that laser shock can also induce local work function modulation in graphene/Cu morphologies beyond the protrusion geometry. Scale bars, 1 μm.

Tables

Table S1. Grouped peak fitting results for the high-resolution C 1s and O 1s XPS spectra.

Values are given as relative fitted area (%), with fitted peak positions in eV shown in parentheses. The relative fitted area was normalized to the total fitted area of the corresponding C 1s or O 1s envelope for each sample, and the values describe the relative distribution of fitted components within each core level. For C 1s, the oxygen-containing C group combines the fitted components assigned to C–O, C=O and O–C=O related carbon contributions. For O 1s, the O in oxygen-containing C group combines the fitted oxygen components assigned to C=O/O–C=O and C–O contributions. The low-BE O component is assigned to strongly bound or subsurface oxygen species.

Sample	C 1s			O 1s		
	**sp^2 C**	**sp^3 C**	**oxygen-containing C**	**low-BE O**	**Cu–O**	**O in oxygen-containing C**
1-p	76.2 (284.50)	15.4 (285.38)	8.4 (286.80, 288.59)	11.4 (528.12)	69.7 (530.48)	18.9 (532.03)
1-L	65.6 (284.55)	20.3 (285.74)	14.1 (287.47, 288.98)	2.8 (528.15)	61.1 (530.49)	36.1 (532.01)
2-p	70.4 (284.57)	20.4 (285.34)	9.2 (286.89, 288.66)	12.7 (527.92)	46.5 (530.83)	40.8 (532.24)
2-L	54.1 (284.51)	26.5 (285.35)	19.5 (286.60, 288.44)	—	38.8 (530.65)	61.2 (531.91, 533.18)

Table S2. Simplified DFT models for comparing representative factors affecting the work function.

Laser shock produces coupled changes in the graphene/Cu surface and interface. These simplified models were constructed to compare the effects of representative structural and interfacial changes identified from the experimental observations on the work function.

Model	Simplified structure	Experimentally motivated factor	Work function (eV)
M1	Monolayer graphene/Cu(111)	Baseline monolayer graphene/Cu interface	4.907
M2	Monolayer graphene/Cu(111) with a graphene vacancy	Graphene defect (Raman D and D′ bands and local graphene discontinuity after laser shock)	4.922
M3	Monolayer graphene/Cu(111) with a C–O bridge configuration	Oxygenated functionality (XPS evidence of oxygen-containing carbon species after laser shock)	4.891
M4	Monolayer graphene on stepped Cu(211)	Stepped Cu surface configuration (strong Cu deformation and local structural heterogeneity near the protrusion sidewall and root regions)	4.429
M5	Bilayer graphene/Cu(111)	Baseline bilayer graphene/Cu interface	4.889
M6	Bilayer graphene/Cu(111) with increased graphene/Cu spacing	Effective interfacial decoupling (possible interlayer sliding and oxygen intercalation after laser shock)	4.786

References

1. R. Fabbro, J. Fournier, P. Ballard, D. Devaux, and J. Virmont, "Physical Study of Laser-Produced Plasma in Confined Geometry," *Journal of Applied Physics* 68, no. 2 (1990): 775–784. https://doi.org/10.1063/1.346783
2. A. Rondepierre, A. Sollier, L. Videau, and L. Berthe, "Review on Laser Interaction in Confined Regime: Discussion about the Plasma Source Term for Laser Shock Applications and Simulations," *Metals* 11, no. 12 (2021): 2032. https://doi.org/10.3390/met11122032
3. H. Kuttruff, *Acoustics: An Introduction* (London: Taylor & Francis, 2007).
4. W. Zhang and Y. L. Yao, "Micro Scale Laser Shock Processing of Metallic Components," *Journal of Manufacturing Science and Engineering* 124, no. 2 (2002): 369–378. https://doi.org/10.1115/1.1445149
5. J. E. Lee, G. Ahn, J. Shim, Y. S. Lee, and S. Ryu, "Optical Separation of Mechanical Strain from Charge Doping in Graphene," *Nature Communications* 3, no. 1 (2012): 1024. https://doi.org/10.1038/ncomms2022
6. G. Ahn and S. Ryu, "Reversible Sulfuric Acid Doping of Graphene Probed by In-Situ Multi-Wavelength Raman Spectroscopy," *Carbon* 138 (2018): 257–263. https://doi.org/10.1016/j.carbon.2018.05.065
7. K. Kumagai and T. Sekiguchi, "Sharing of Secondary Electrons by In-Lens and Out-Lens Detector in Low-Voltage Scanning Electron Microscope Equipped with Immersion Lens," *Ultramicroscopy* 109, no. 4 (2009): 368–372. https://doi.org/10.1016/j.ultramic.2009.01.005
8. B. J. Griffin, "A Comparison of Conventional Everhart-Thornley Style and In-Lens Secondary Electron Detectors—A Further Variable in Scanning Electron Microscopy," *Scanning* 33, no. 3 (2011): 162–173. https://doi.org/10.1002/sca.20255
9. H. Seiler, "Secondary Electron Emission in the Scanning Electron Microscope," *Journal of Applied Physics* 54, no. 11 (1983): R1–R18. https://doi.org/10.1063/1.332840
10. M. Sezgin and B. Sankur, "Survey over Image Thresholding Techniques and Quantitative Performance Evaluation," *Journal of Electronic Imaging* 13, no. 1 (2004): 146–168. https://doi.org/10.1117/1.1631315
11. V. Maier-Kiener and K. Durst, "Advanced Nanoindentation Testing for Studying Strain-Rate Sensitivity and Activation Volume," *JOM* 69, no. 11 (2017): 2246–2255. https://doi.org/10.1007/s11837-017-2536-y
12. W.-G. Jiang, J.-J. Su, and X.-Q. Feng, "Effect of Surface Roughness on Nanoindentation Test of Thin Films," *Engineering Fracture Mechanics* 75, no. 17 (2008): 4965–4972. https://doi.org/10.1016/j.engfracmech.2008.06.016
13. W. Melitz, J. Shen, A. C. Kummel, and S. Lee, "Kelvin Probe Force Microscopy and Its Application," *Surface Science Reports* 66, no. 1 (2011): 1–27. https://doi.org/10.1016/j.surfrep.2010.10.001
